# A Comprehensive Meta‐Analysis of Bioadaptor Versus Drug‐Eluting Stents in Randomised Trials With Exploratory Single‐Arm Landmark Analyses

**DOI:** 10.1111/eci.70217

**Published:** 2026-05-05

**Authors:** Simon Wölbert, Stephanie Kühne, Andrea Patrignani, Mauro Chiarito, Moritz von Scheidt, Jan Torzewski, Philip Raake, Dario Bongiovanni

**Affiliations:** ^1^ Department of Internal Medicine I, Cardiology University Hospital Augsburg, University of Augsburg Augsburg Germany; ^2^ Department of Biomedical Sciences Humanitas University Milan Italy; ^3^ Humanitas Research Hospital IRCCS Milano Italy; ^4^ Department of Cardiology German Heart Center, TUM University Hospital, Technical University Munich Munich Germany; ^5^ German Center for Cardiovascular Research (DZHK), Partner Site Munich Heart Alliance Munich Germany; ^6^ Cardiovascular Center Oberallgäu‐Kempten Kempten Germany

**Keywords:** bioadaptor, drug‐eluting stents, meta‐analysis, percutaneous coronary intervention, randomised controlled trials, target‐lesion failure

## Abstract

**Background:**

Late adverse events after percutaneous coronary intervention continue to occur beyond the first year with last‐generation drug‐eluting stents (DES). The coronary bioadaptor marks a new approach with an uncaging beginning at approximately 6 months after implantation. We conducted a pairwise meta‐analysis of bioadaptor versus DES in randomised trials with complementary single‐arm 6–12 and 6–24 landmark analyses.

**Methods:**

The systematic review and meta‐analysis was conducted according to PRISMA 2020 Guidelines. PubMed, Embase, CENTRAL and Google Scholar were searched for studies reporting clinical outcomes after bioadaptor implantation. The primary outcome was target‐lesion failure (TLF), a composite of cardiac death, target‐vessel myocardial infarction and target‐lesion revascularisation at 1 year. Secondary outcomes were TLF and individual components at landmark intervals 6–12 and 6–24 months. Single‐arm pooled event rates and pairwise comparisons were estimated using generalised linear mixed‐effects models.

**Results:**

Three randomised trials (*n* = 2892; 1448 bioadaptor, 1444 DES) were included in pairwise analyses. 1‐year TLF showed no significant difference between bioadaptor and DES (OR 0.81, 95% CI 0.51–1.31, *I* ^2^ = 0.0%, *p* = 0.3943). Likewise, individual components of TLF and device thrombosis did not differ between groups. Ten studies (1753 patients; 1900 lesions) were included in single‐arm analyses. Landmark TLF was 0.57% (95% CI 0.07–4.29; *I* ^2^ = 4.6%) from 6 to 12 months and 2.01% (95% CI 0.81–4.92; *I* ^2^ = 74.2%) from 6 to 24 months. Event rates for other endpoints were generally low.

**Conclusions:**

No significant differences in safety and efficacy outcomes were observed between bioadaptor and DES. Complementary single‐arm landmark analyses suggested low late event rates, but these findings should be interpreted as exploratory. Further randomised trials are warranted.

## Graphical abstract

This meta‐analysis evaluated clinical outcomes following coronary bioadaptor implantation compared with drug‐eluting stents. Pairwise analysis of randomised trials showed no significant difference in target‐lesion failure at 1 year. Complementary single‐arm landmark analyses demonstrated low late event rates between 6 and 12 months and 6 and 24 months. These findings suggest favourable late safety and efficacy of the bioadaptor, although further adequately powered randomised trials are required to confirm these results.

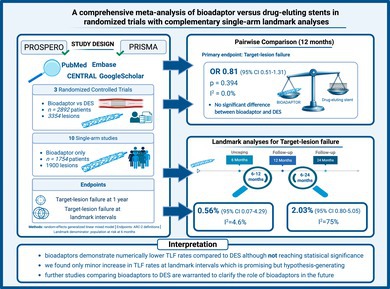

**Abbreviations**
ACS: acute coronary syndrome
BA: bioadaptor
CD: cardiac death
CD‐TLR: clinically‐driven target‐lesion revascularisation
DES: drug‐eluting stent
MI: myocardial infarction
PCI: percutaneous coronary intervention
STEMI: ST‐segment elevation myocardial infarction
TLF: target‐lesion failure
TVMI: target‐vessel myocardial infarction

## 1 Introduction

Percutaneous coronary intervention (PCI) has undergone continuous technical improvement from the development of drug‐eluting stents (DES) in the early 2000s [1]. A landmark analysis of the BIO‐RESORT randomised controlled trial (RCT) comparing contemporary DES showed favourable outcomes at 1 year, however, adverse events accrue 1–5 years following PCI. This has also been confirmed in several meta‐analyses [2, 3]. To overcome this limitation, bioresorbable scaffolds and bioresorbable vascular scaffolds (BRS, e.g., Magmaris; BVS, e.g., Absorb) have been developed. The hypothesis was that temporary vessel support followed by bioresorption could reduce late stent‐related adverse events by limiting neointimal hyperplasia and neoatherosclerosis. Both BRS achieved promising results by liberating stented coronary artery segments, leading to restoration of vasomotion, late lumen enlargement and physiological arterial healing [4, 5, 6]. Nevertheless, these technologies failed to provide any benefits revealing an increased risk for scaffold thrombosis [7]. To address this unmet need, the DynamX sirolimus‐eluting coronary bioadaptor (BA; Elixir Medical Corporation, Milpitas, CA) was developed. This platform is made from three 71‐μm cobalt‐chromium (CoCr) sinusoidal helical strands connected by three unlocking elements per ring along the stent. The elements are covered by a bioresorbable polymer. After 6 months, the polymer layer is resorbed, enabling the CoCr helical strands to initiate the unlocking mechanism. As strands separate, the stent uncages the vessel, restoring motion and physiologic function while maintaining dynamic mechanical support accommodating vessel biomechanics [8]. It remains uncertain whether BA can achieve comparable clinical outcomes to DES, particularly beyond the early post‐implantation phase. We therefore conducted a comprehensive systematic review and meta‐analysis of RCTs with an exploratory single‐arm (SA) analysis to synthesise current evidence on landmark data.

## 2 Methods

This systematic review and meta‐analysis was conducted according to recommendations of the Cochrane Collaboration Handbook for Systematic Reviews of Interventions, and we adhered to the most recent version of Preferred Reporting Items for Systematic Reviews and Meta‐Analyses (PRISMA) guidelines [9, 10]. The review protocol was prospectively registered in the International Prospective Register of Systematic Reviews (PROSPERO; CRD 420251235741).

### 2.1 Eligibility Criteria

We identified and included studies that fulfilled the following predefined criteria: (1) implantation of at least one DynamX BA; (2) in patients with symptomatic coronary artery disease; (3) reported at least one outcome of interest; (4) provided sufficient baseline information on the study population (baseline and lesion characteristics). We excluded publications meeting any of the following criteria: (1) case reports and case series with fewer than 20 patients; (2) preclinical, in vitro or animal studies; (3) registries lacking baseline or outcome information relevant to the predefined endpoints.

### 2.2 Search Strategy and Data Extraction

PubMed, Embase via Ovid, CENTRAL via Ovid and Google Scholar were systematically searched for eligible studies with a time restriction from 1 January 2017 to 31 January 2026. We did not apply any language restriction. Search strategies combined database‐specific indexing terms (MeSH and Emtree, where applicable) with free‐text keywords referring to the DynamX bioadaptor device and its proprietary nomenclature (e.g., ‘DynamX bioadaptor’, ‘coronary bioadaptor system’, ‘sirolimus‐eluting bioadaptor’, ‘Elixir DynamX’). The search syntax was adapted to the structure of each database, and title/abstract fields were used where controlled vocabulary was not available. A detailed search strategy for each database is provided in Appendix S1. To make sure that no relevant reports were overlooked, reference lists of included studies were screened (backwards snowballing). Literature search and data extraction was independently performed by the first author and S.G.K. while disagreements were solved by consulting a third scientist (D.B.).

Baseline clinical characteristics included age, sex, clinical presentation (acute coronary syndrome (ACS)) and common cardiovascular risk factors such as diabetes mellitus (DM), arterial hypertension (HT), smoking and prior myocardial infarction. Procedural characteristics comprised lesion complexity, lesion length, reference vessel diameter, target vessel and degree of calcification.

If landmark outcomes were not reported directly in the original publication, event counts were reconstructed from the available study data. This included Kaplan–Meier curves, reported landmark percentages or descriptions of interval‐specific events (see Appendix S1 for detailed information). For landmark analyses starting at 6 months, the number of patients at risk at 6 months was used as the denominator. If only percentages were reported, absolute numbers were estimated by multiplying the reported percentage by the corresponding 6‐month risk set and rounding to the nearest whole number. Consequently, event numbers for the 6–12‐month and 6–24‐month intervals refer to patients who remained event‐free up to the 6‐month follow‐up. Reconstructed values were treated as estimates rather than directly reported counts and were interpreted accordingly, acknowledging the assumptions inherent to Kaplan–Meier‐based reconstruction, including censoring patterns. This approach is commonly used when no access to individual patient data is granted [11].

### 2.3 Quality Assessment

Risk of Bias assessment for single‐arm studies was performed according to Joanna Briggs Institute (JBI) Critical Appraisal Checklist for Case Series evaluating 10 domains addressing study selection, measurement validity, outcome reporting and statistical analysis [12]. Key methodological domains were clarity of eligibility criteria, completeness and consecutiveness of patient inclusion, adequacy of follow‐up, and appropriateness of the statistical analyses. Studies meeting at least eight domains without major methodological shortcomings were classified as low risk of bias. Studies with minor limitations but no major concerns were judged as having some concerns, whereas studies with one or more major methodological shortcomings were classified as high risk of bias. RCTs were evaluated according to Cochrane's Risk of Bias‐2 tool (RoB‐2) [13]. Risk of bias assessment was independently performed by two authors (S.W. and S.G.K.) and disagreements were solved by consulting the corresponding author (D.B.).

### 2.4 Outcome Measures

The primary outcome measure was target‐lesion failure (TLF), defined as a composite of cardiac death (CD), target‐vessel myocardial infarction (TVMI) and clinically‐driven target‐lesion revascularisation (CD‐TLR) at 12 months according to the definition of the Academic Research Consortium‐2 (ARC‐2) [14]. Secondary endpoints comprised TLF and individual components of TLF at landmark intervals 6–12 months, 6–24 months and at 24 months follow‐up.

### 2.5 Statistical Analysis

Baseline characteristics are reported per each study included, with means and standard deviation or median and interquartile ranges for continuous variables and percentages for categorical variables. Mean weighted baseline characteristics were derived using study‐level estimates weighted by the number of patients or lesions contributing to each variable, with pooled values obtained by normalisation to the total sample size. Meta‐analyses were conducted using a random‐effects generalised linear mixed‐effects model. To compare clinical outcomes between BA and DES, odds ratios (OR) with 95% confidence intervals were estimated, and *p* values were calculated to assess statistical differences between groups. For each outcome, study‐specific event proportions were calculated and pooled to obtain overall event rates with 95% confidence intervals. This approach was chosen due to its generally superior performance compared to conventional two‐step methods for meta‐analyses of proportions [15]. Heterogeneity was assessed by *I* ^2^ statistics and the Wald test for heterogeneity. We interpreted *I* ^2^ according to Cochrane recommendations (low: 0%–40%, moderate: 30%–60%, substantial: 50%–90%, considerable: 75%–100%) [10]. Moderate, substantial or considerable heterogeneity was explored by the following methods: first, forest plots were visually inspected to identify potential outlying studies. For each outlier, the original report was reviewed in detail to assess clinical and methodological features that differed from the remaining cohorts (e.g., patient risk profile, lesion complexity). Second, sensitivity analyses using the leave‐one‐out (LOO) method were performed for primary outcomes where appropriate. Third, we planned a prespecified analysis where studies published only as conference abstracts were excluded to account for incomplete reporting and potential publication bias associated with non‐peer‐reviewed sources. Last, a meta‐regression analysis for prespecified study‐level moderators (age, DM, ACS) was performed to investigate their potential influence on outcomes with moderate heterogeneity. Statistical analyses were performed using R version 4.5.1 (R Foundation for Statistical Computing, Vienna, Austria), with RStudio used as the integrated development environment (Posit team (2025). RStudio: Integrated Development Environment for R. Posit Software, PBC, Boston, MA).

## 3 Results

### 3.1 Study Selection and Baseline Characteristics

**FIGURE 1:**
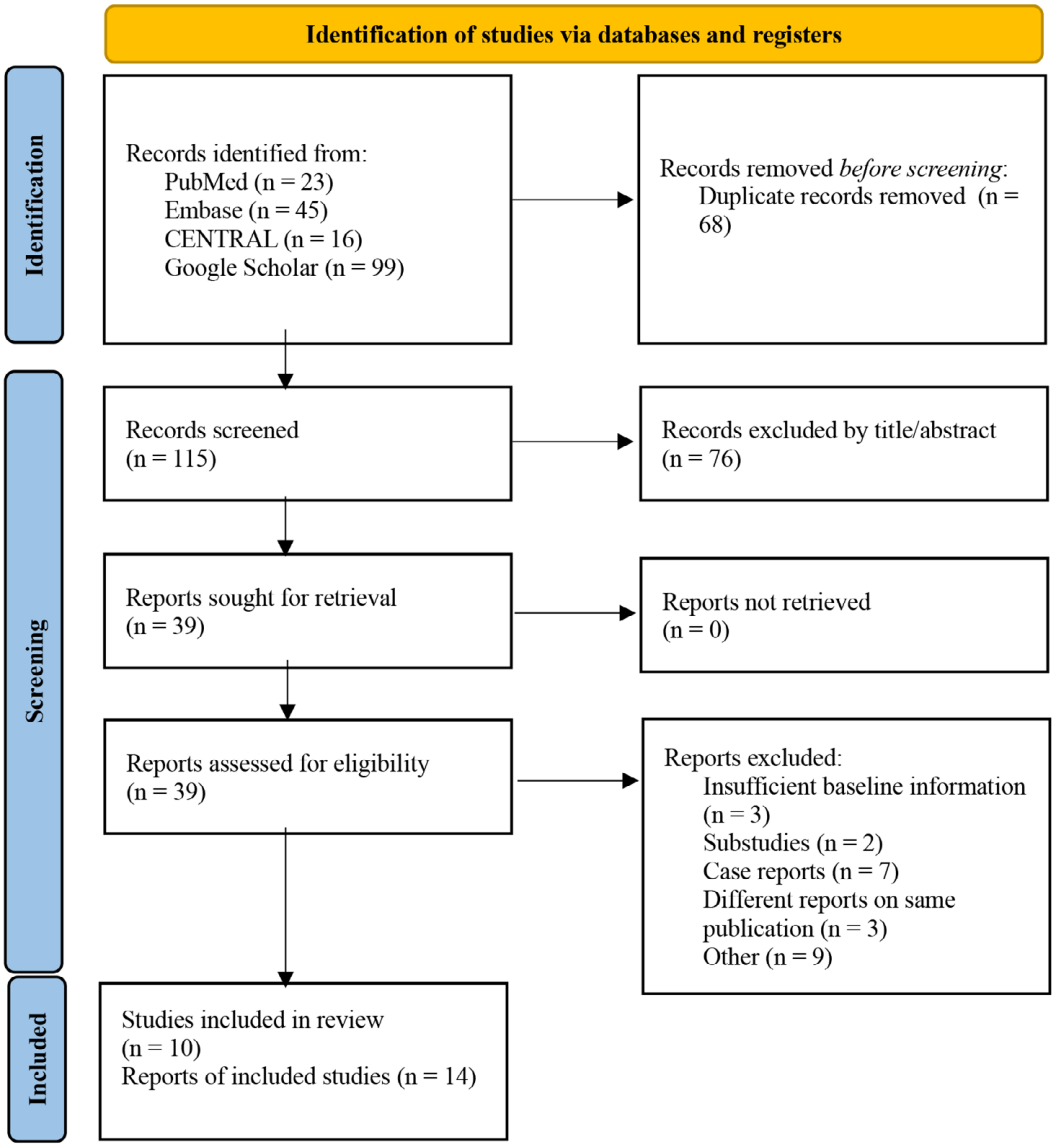
PRISMA 2020 flow diagram of study identification, screening, eligibility assessment and inclusion.

The initial search strategy yielded 186 results. After removal of duplicates and screening 38 reports (full‐text review), we considered three RCTs eligible for pairwise analysis comprising 2892 patients (*n* = 1448 BA, *n* = 1444 DES) with 3354 lesions (*n* = 1669 BA; *n* = 1685 DES). For SA analyses, 10 studies encompassing a total of 1753 patients and 1900 lesions (two studies [16, 17] did not report the exact number of lesions) were included (Figure 1; Table S1) [8, 16, 17, 18, 19, 20, 21, 22, 23, 24]. Baseline clinical characteristics were comparable between groups with mean weighted age 69.2 vs. 68.7 years, male sex 76.4% vs. 76.7% and ACS at presentation 66.0% vs. 64.8% for BA and DES, respectively. Nearly all patients completed 12‐month follow‐up (Table 1, Table S2). Lesions were 23.20 mm vs. 23.53 mm long, 40.1% and 38.7% of lesions were type B2/C, in BA and DES, respectively. In both groups, approximately 50% of lesions were located in the left anterior descending artery (LAD) (Table 2, Table S3). In SA analysis, mean weighted age was 68.9 years, approximately 77% were male. 64.9% of patients presented with ACS; DM and HT were present in 22.8% and 61.8%, respectively (Table 3, Table S4). Four out of 10 included studies were conference abstracts [16, 17, 21, 23], and therefore, sufficient information for treated lesions was not consistently available. Seven studies [8, 18, 19, 20, 21, 22, 24] reported the proportion of Type B2/C lesions (weighted % 40.5; *n* = 1875), six studies [8, 18, 19, 20, 21, 24] described lesion length (mean weighted length 22.6 mm; *n* = 1820), and eight studies [8, 18, 19, 20, 21, 22, 23, 24] mentioned proportion of left anterior descending artery (LAD) as the treated target vessel (weighted % 51.1; *n* = 1900) (Table 4, Table S5).

**TABLE 1:** Baseline clinical characteristics of randomised controlled trials.

Study BA/DES	Saito, 2023 [19] DynamX/Onyx	Erlinge, 2024 [18] DynamX/Onyx	Tsai, 2026 [24] DynamX/DESyne
Patients (*n*)	223/222	1201/1198	24/24
Age (y)	67.1 (10.3)/66.2 (10.1)	69.7 (61.2–75.7)/69.3 (61.5–75.6)^a^	64.38 (9.59)/62.83 (7.86)
Male (%)	78.0/77.9	75.9/76.2	87.5/87.5
ACS (%)	13.9/8.6	77.0/76.2	4.2/16.7
Smoking history (%)	66.2/65.4	14.2/16.1	29.2/33.3
Diabetes mellitus (%)	26.5/33.8	19.3/16.6	45.8/37.5
Hypertension (%)	73.2/70.9	60.5/59.9	62.5/45.8
Dyslipidemia (%)	80.9/80.5	45.6/41.5	91.7/95.8
Previous PCI (%)	40.0/37.7	14.7/13.9	29.2/45.8
Previous CABG (%)	0.9/0.5	1.0/0.7	0.0/4.2
Multivessel disease (%)	9.9/12.7	10.8/12.1	91.7/83.3
12‐month follow‐up (%)	99.1/96.8	98.5/99.0	100/100

*Note:* Data are presented as percentages or mean (SD), unless otherwise indicated.

Abbreviations: ACS, acute coronary syndrome; CABG, coronary arterial bypass graft; *n*, number of patients; PCI, percutaneous coronary intervention; y, years.

^a^ Median with interquartile range.

**TABLE 2:** Baseline lesion characteristics of randomised controlled trials.

Study	Saito, 2023 [19]	Erlinge, 2024 [18]	Tsai, 2026 [24]
Lesions (*n*)	226/230	1419/1431	24/24
Type B2/C (%)	22.6/21.3	43.3/41.4	20.8/45.9
Diameter stenosis (%)	64.39 ± 11.04/64.40 ± 13.29	87.0 ± 12.1/87.3 ± 11.7	77.88 ± 9.10/83.29 ± 11.12
Reference diameter (SD)	3.1 (0.4)/3.0 (0.4)	3.2 (0.5)/3.2 (0.5)	3.15 (0.42)/3.07 (0.35)
Lesion length (SD)	15.8 (6.0)/16.2 (6.0)	24.4 (9.1)/24.7 (9.4)	21.71 (7.04)/23.71 (6.23)
Target vessel LAD (%)	50.4/54.2	51.2/50.9	45.8/25.0
Target vessel RCA (%)	34.1/26.1	23.0/22.2	25.0/41.7
Target vessel LCX (%)	15.5/28.7	25.7/26.8	29.2/33.3
Bifurcation (%)	22.1/23.9	11.8/11.3	NA
Calcification moderate/severe (%)	19.0/20.4	16.9/14.3	NA
Tortuosity moderate/severe (%)	23.5/20.0	7.7/7.3	NA
Predilatation (%)	100/100	99.7/99.9	100/100
Postdilatation (%)	80.1/69.1	58.5/52.9	100/95.8

*Note:* Data are presented as percentages or mean (SD), unless otherwise indicated.

Abbreviations: LAD, left anterior descending artery; LCX, left circumflex artery; *n*, number of lesions; RCA, right coronary artery; SD, standard deviation.

**TABLE 3:** Baseline clinical characteristics of observational studies.

Study	*n*	Age (y)	Male (%)	ACS (%)	Smoking (%)	DM (%)	HT (%)	Prior MI (%)	12m FU (%)
Verheye, 2020 [8]	50	66.3	74	2	76	26	70	30	92
Yan, 2023 [21]	49	64.0	82	52	30	36	78	NA	100
Webster, 2024 [20]	44	61.7	75	68	64	18	54	25	97.8
Leone, 2026 [22]	55	67	92.7	14.5	NA	29.1	NA	25.4	100
Ho, 2025 [16]	40	56.4	82.5	100	NA	15	NA	NA	NA
Kang, 2025 [23]	25	55.8	72	100	16	40	28	NA	100
Wong, 2025 [17]	42	58	93	100	64	26	33	0	95.2

*Note:* Data are reported as indicated.

Abbreviations: 12m FU, 12‐month follow‐up completed; ACS, acute coronary syndrome; DM, diabetes mellitus; HT, arterial hypertension; MI, myocardial infarction; *n*, number of patients; NA, not available; *y*, years.

**TABLE 4:** Baseline lesion characteristics of observational studies.

Study	*n*	Type B2/C (%)	Lesion length (mm)	RD (mm)	TV LAD (%)	BA length (mm)	BA diameter (mm)
Verheye, 2020 [8]	50	50	11.1 ± 5.1	2.91 ± 0.43	44	NA	NA
Yan, 2023 [21]	56	11	19.5 ± 6.6	3.05 ± 0.45	46	NA	NA
Webster, 2024 [20]	45	7	16.0 ± 6.7	2.81 ± 0.41	53	NA	3.1
Leone, 2026 [22]	55	100	NA	NA	52.7	28 ± 7	3.2 ± 0.3
Ho, 2025 [16]	NA	NA	NA	NA	NA	28.6 ± 7.6	3.1 ± 0.4
Kang, 2025 [23]	25	NA	NA	NA	72	26.8	2.68
Wong, 2025 [17]	NA	NA	NA	NA	71.4	35	3.3

*Note:* Data are reported as numbers, percentages (%) or mean ± standard deviation.

Abbreviations: BA, bioadaptor; LAD, left anterior descending artery; *n*, number of lesions; NA, not available; TV, target‐vessel.

### 3.2 1‐Year Outcomes of Bioadaptor Versus Drug‐Eluting Stent

**FIGURE 2:**
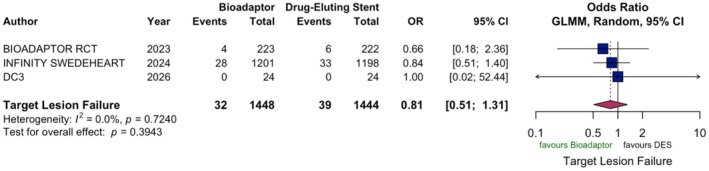
Pairwise meta‐analysis of primary outcome target‐lesion failure at 1 year. Odds ratios (OR) with 95% confidence intervals (CI) for target‐lesion failure comparing bioadaptor with drug‐eluting stents were calculated. A random‐effects generalised linear mixed effects model (GLMM) was applied to estimate pooled effects. Heterogeneity was assessed using the *I* ^2^ statistic.

For target lesion failure at 1 year, the random‐effects pairwise meta‐analysis showed no significant difference between BA and DES (OR 0.81, 95% CI 0.51–1.31; *p* = 0.394, Figure 2). At 1 year, CD rates were comparable between BA and DES (OR 0.87, 95% CI 0.32–2.41; *p* = 0.792). Similarly, no difference was observed for TVMI (OR 0.81, 95% CI 0.43–1.52; *p* = 0.519), CD‐TLR (OR 0.65, 95% CI 0.35–1.20; *p* = 0.167) and DT (OR 1.28, 95% CI 0.48–3.46; *p* = 0.6209) (Figure S1A–D). Notably, there was no between study heterogeneity in all outcomes (*I* ^2^ = 0.0%).

### 3.3 Outcomes for Landmark Analysis

**FIGURE 3:**
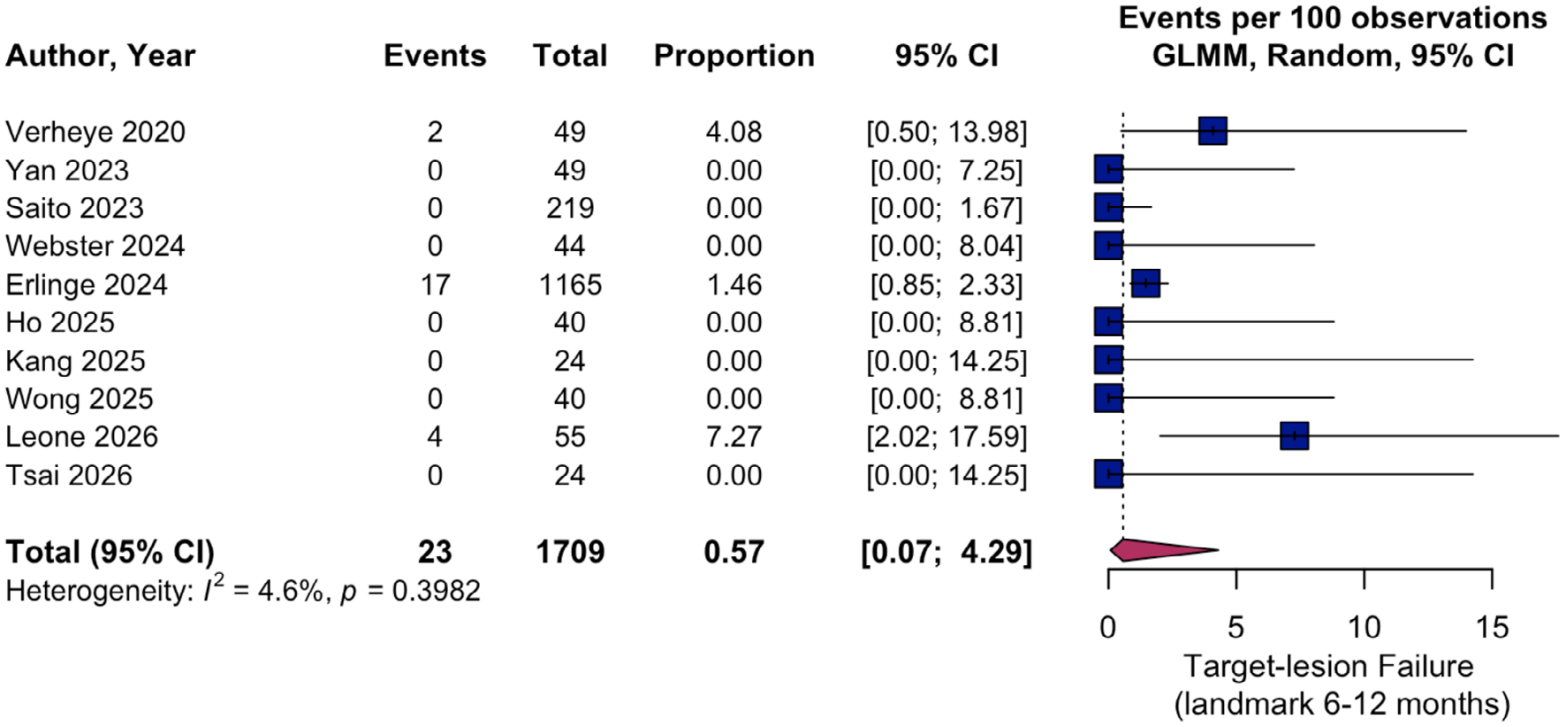
Single‐arm meta‐analysis of secondary outcome target‐lesion failure at landmark interval 6–12 months. Pooled event rates with 95% confidence intervals (CI) for target‐lesion failure at landmark interval 6–12 months are shown. GLMM, generalised linear mixed effects model.

Sufficient data for landmark analysis between 6 and 12 months were directly reported or could be reliably reconstructed for all studies, whereas landmark analysis between 6 and 24 months was available only in four studies [8, 18, 19, 22]. Overall, event rates for the secondary outcome TLF and composites between 6 and 12 months were low. There was little heterogeneity for TLF and TVMI and substantial heterogeneity for CD‐TLR. Pooled event rate for TLF was 0.57% (95% CI 0.07–4.29; *I* ^2^ = 4.6%; Figure 3), CD‐TLR showed a pooled incidence of 0.27% (95% CI 0.03–2.33; *I* ^2^ = 60.1%) and TVMI yielded a pooled proportion of 0.18% (95% CI 0.04–0.88; *I* ^2^ = 0.0%) (Figures S2 and S3). Between 6 and 12 months, only 2 CD occurred among 1712 patients at risk; 9 of 10 cohorts had no events. Therefore, no formal statistical analysis using the GLMM approach was conducted for cardiac mortality.

**FIGURE 4:**
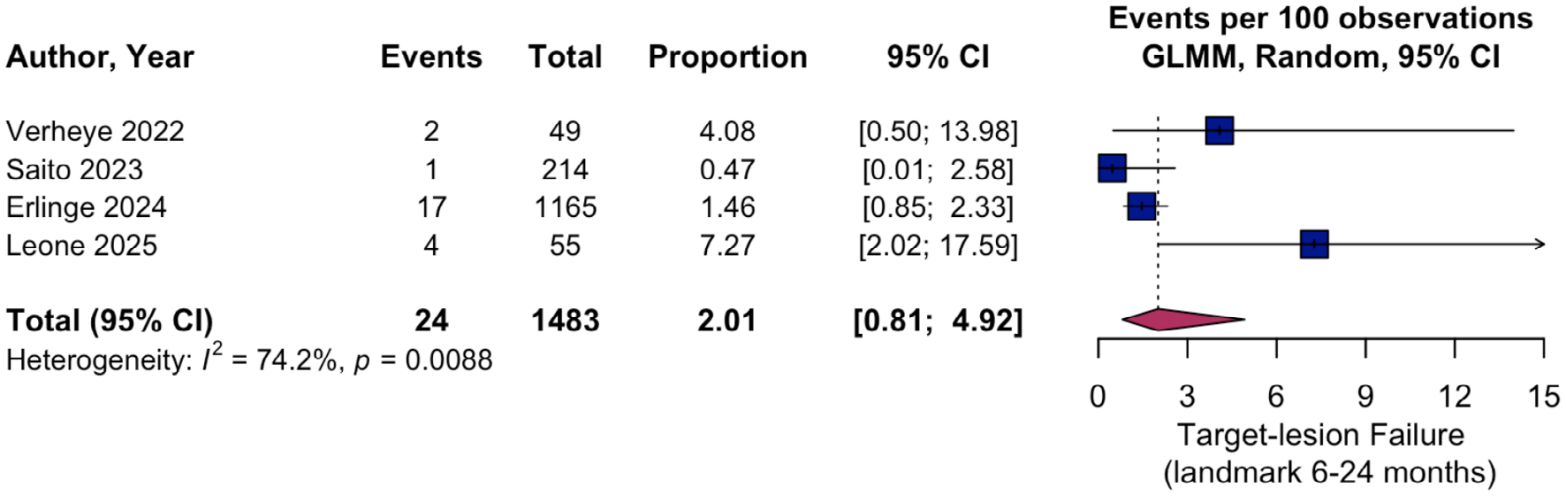
Single‐arm meta‐analysis of secondary outcome target‐lesion failure at landmark interval 6–24 months. Pooled event rates per 100 observations with 95% confidence intervals (CI) for target‐lesion failure at landmark interval 6–24 months are shown. GLMM, generalised linear mixed effects model.

Between 6 and 24 months, pooled TLF rate was 2.01% (95% CI 0.81–4.92; *I* ^2^ = 74.2%; Figure 4), CD was 0.35% (95% CI 0.05–2.20; *I* ^2^ = 67.3%), CD‐TLR was 0.82% (95% CI 0.18–3.76; *I* ^2^ = 84.3%) and TVMI showed a pooled rate of 0.35% (95% CI 0.05–2.20; *I* ^2^ = 67.3%; Figures S4–S6).

### 3.4 Outcomes at 12 and 24 Months

**FIGURE 5:**
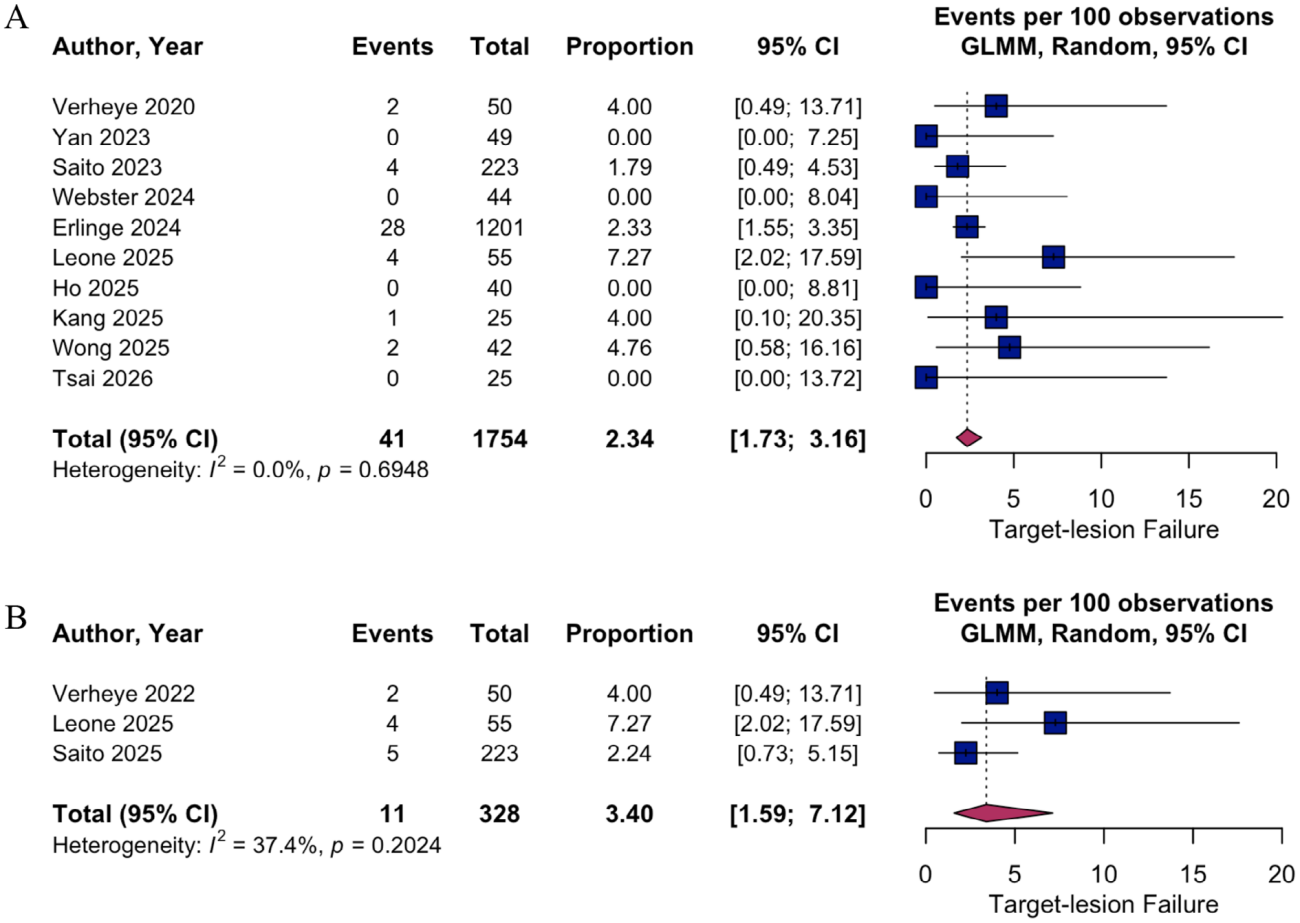
Single‐arm meta‐analysis of primary outcome target‐lesion failure at 12 and secondary outcome at 24 months. Pooled event rates per 100 observations with 95% confidence intervals (CI) for target‐lesion failure at 12 (A) and 24 months (B) are shown. GLMM, generalised linear mixed effects model.

For TLF at 12 months, the pooled event rate was 2.34% (95% CI 1.73–3.16; *I* ^2^ = 0.0%; Figure 5A). CD yielded an incidence of 0.53% (95% CI 0.12–2.26; *I* ^2^ = 13.7%), TVMI showed 1.14% (95% CI 0.74–1.76; *I* ^2^ = 0.0%), and CD‐TLR was 1.07% (95% CI 0.37–3.02; *I* ^2^ = 21.7%) at 12‐month follow‐up (Figures S7–S9). 24‐month follow‐up was available for three studies [22, 25, 26] including 328 individuals with a total of 11 events reported. The estimated incidence of TLF was 3.40% (95% CI 1.59–7.12; *I* ^2^ = 37.4%; Figure 5B).

### 3.5 Sensitivity Analyses

LOO analysis for CD‐TLR landmark data 6–12 months showed that there was no residual heterogeneity when Leone 2026 and Erlinge 2024 were omitted, that is, *I* ^2^ was reduced from 60.1% to 0.0%. Overall pooled incidences did not change significantly (Figure S10). By performing LOO analysis for the TLF landmark data 6–24 months, heterogeneity was decreased from *I* ^2^ = 75% to *I* ^2^ = 41.4% by omitting Leone 2026 (Figure S11). In leave‐one‐out sensitivity analysis for CD‐TLR landmark data 6–24 months, omission of Verheye 2022, Saito 2025 or Erlinge 2025 resulted in only minor changes in the pooled landmark TLR estimate (range 1.03%–1.10%). In contrast, exclusion of Leone 2026 reduced the pooled estimate from 0.82% to 0.42% and eliminated between‐study heterogeneity (*I* ^2^ from 67.9%–89.5% to 0%), indicating that Leone 2026 was the main contributor to heterogeneity and had the greatest influence on the pooled estimate (Figure S12). Sensitivity analyses excluding studies published as abstracts only yielded consistent results. The pooled incidence of target‐lesion failure between 6 and 12 months was 1.2 events per 100 observations (95% CI, 0.28–4.84), and clinically driven target‐lesion revascularisation was 0.24 events per 100 observations (95% CI, 0.01–3.76), supporting consistency of the primary findings (Figure S13A,B).

### 3.6 Meta‐Regression Analyses and Quality of Studies

Meta‐regression analyses were deemed appropriate for investigation of the low to moderate heterogeneity reported for CD‐TLR (*I* ^2^ = 21.7%) at 12 months. None of the clinical study‐level covariates (age, proportion of DM, proportion of ACS) showed a relevant association with 12‐month CD‐TLR (Figure S14A–C). Assessment of relevant lesion‐level characteristics was not feasible since lesion properties were inconsistently reported (see Section 3.1).

For SA studies, overall study quality was heterogeneous. Four studies [8, 20, 21, 22] were assessed as low risk of bias, one study [16] showed some concerns, and two studies [17, 23] were classified as high risk of bias, mainly because of incomplete reporting of patient inclusion, follow‐up procedures and outcome assessment (Table S6). Risk of Bias was considered low for RCTs (Table S7).

## 4 Discussion

To the best of our knowledge, this is the first pairwise and SA meta‐analysis primarily investigating landmark analysis following BA implantation in patients with coronary artery disease. The main findings of our study were: (1) No significant differences in safety and efficacy were observed between BA and DES; (2) TLF between 6 and 12 months after PCI with the BA was very low (0.57%), with negligible heterogeneity for TLF and TVMI and only substantial heterogeneity for CD‐TLR; (3) between 6 and 24 months, cumulative TLF incidence increased to 2.01%; (4) pooled 12‐ and 24‐month incidences of TLF were 2.34% and 3.40%, respectively, with overall low rates of cardiac death and TVMI.

Results of our pairwise meta‐analysis showed no statistically significant differences in safety and efficacy outcomes between BA and DES. TLF and its individual components were numerically lower with BA, while between‐study heterogeneity was absent across randomised trials. Mechanistic studies suggest that BA can restore radial and torsional motion as well as cyclic pulsatility. These device characteristics were associated with positive adaptive remodelling and thus may reduce neointimal hyperplasia and neoatherosclerosis [8]. In the BIOADAPTOR‐RCT, the authors conducted imaging sub‐studies and found regression of atherosclerotic plaques in coronary segments stented with BA. This could not be seen with DES in the same study [19]. Together, these characteristics may enable coronary arteries stented with BA to achieve late lumen enlargement and thus maintain a stable lumen, even 1 year after PCI [20]. However, whether these mechanistic observations translate into lower rates of late adverse clinical events remains to be confirmed during longer term follow‐up. Nevertheless, only three RCTs were available for pairwise comparison, limiting the precision of the pooled estimates for infrequent clinical events such as CD and DT.

The quality of included observational SA studies was inconsistent, and limitations in reporting and follow‐up in some cohorts may have influenced the precision of pooled outcomes. In contrast, the RCTs demonstrated a low risk of bias and provided methodologically robust evidence.

Critical time intervals following contemporary DES implantations are considered to be 0–30 days and beyond 1 year and thus, specific 6–12 and 6–24 months landmark analyses in DES trials are seldom reported [27]. Ideal comparison of landmark intervals can only be addressed in dedicated RCTs and makes direct comparison of our results to broad literature difficult since the uncaging mechanism at 6 months is unique to BA. Thus, the 6‐month timepoint is mechanistically pivotal for the BA, making 6‐month landmark analyses indispensable to assess clinical performance during the post‐uncaging phase. Of note, event rates observed in the 6–24 month interval appeared numerically low when viewed in the context of published contemporary DES data.

The pooled 12‐month incidence of TLF and its components was low (2.3%) without heterogeneity (0%), suggesting stable performance of the bioadaptor in the first year after implantation. Importantly, the moderate increase (3.4%) in TLF observed at 24 months was driven by a small number of events across three studies and remained well within the range reported for contemporary DES [28]. Given the low absolute event counts and wide confidence intervals, these findings should be interpreted with caution, but they support a sustained and clinically acceptable safety and efficacy profile beyond the first year.

In the BIOSOLVE‐IV registry, investigating the Magmaris‐BRS, event rates were considerably higher: the Kaplan–Meier estimate for TLF increased from approximately 5% at 12 months to 6.8% at 24 months [29]. Between 1 and 2 years, the temporal pattern in BIOSOLVE‐IV therefore differed from that observed in our 6‐ to 12‐ and 6‐ to 24‐month landmark analyses. The successor of Magmaris, Freesolve, a third‐generation BRS with improved mechanical and design features, is currently being tested against Xience in the BIOMAG‐II study and results will show if BRS may regain sufficient attention to compete with BA and DES [30]. Taken together, these observations provide clinical context alongside historical BRS experience, but do not allow comparative interpretation [31].

Although the landmark analyses provide insight into late event patterns following BA implantation, the absence of a concurrent comparator precludes causal inference. Variations in patient risk profiles, lesion characteristics and evolving PCI practices may partly account for the observed outcomes. Therefore, these results should be regarded as exploratory and supportive of mechanistic hypotheses rather than definitive evidence of superiority. These analyses are descriptive in nature and should not be interpreted as comparative evidence against contemporary DES.

Between‐study heterogeneity was moderate to high for CD‐TLR in landmark analysis. LOO sensitivity testing for 6–12 month data indicated that the pooled incidence estimates were stable and not driven by a single study, although heterogeneity resolved when cohorts (Leone 2026; Erlinge 2024) with outlying event rates were omitted. Likewise, heterogeneity resolved for the 6–24 month data when Leone, 2026 was omitted. Notably, the DYNAMITE study also substantially influenced overall pooled for CD‐TLR 6–24 month incidence, which decreased from 0.82 to 0.42 per 100 observations. Inspection of these cohorts suggested that observed heterogeneity was largely driven by differences in the complexity of the study populations. Leone et al. enrolled patients with markedly complex lesions (long lesions, chronic total occlusions, bifurcations, ≥ 4 stents) [22]. Meta‐regression found no association of common clinical covariates (DM, ACS, age) with worse outcomes, underlining the limitations of study‐level analyses in the presence of heterogeneous populations and incomplete lesion‐specific reporting.

Recently, the DynamX Bioadaptor Global Post‐Market Registry: Clinical Trial of the Elixir Medical DynamX Coronary Bioadaptor System (Bio‐RESTORE), NCT06074549, has been initiated and the goal is to include 5000 patients at 17 study sites across Europe, Asia and the Middle East. Despite its SA, open‐label design, results will show whether favourable clinical safety and efficacy can be reproduced in a large all‐comers population.

### 4.1 Limitations

Our study has important limitations. First, we had no access to individual patient data and therefore only study‐level analyses were feasible, limiting the ability to reliably explore potential confounders. Second, four of the 10 studies included in the single‐arm analyses were available only as conference abstracts, which may have introduced reporting bias and limited a comprehensive assessment of study conduct, endpoint adjudication and follow‐up procedures. However, most abstract‐only studies were registered on ClinicalTrials.gov, providing sufficient information on inclusion/exclusion criteria and endpoint definitions, and prespecified sensitivity analyses excluding these studies yielded qualitatively similar results without materially changing the overall interpretation. Third, follow‐up duration varied across studies, and long‐term data beyond 12 months were available only for a limited number of cohorts, restricting conclusions on very late clinical events.

## 5 Conclusion

In this systematic review and meta‐analysis, landmark event rates following DynamX bioadaptor implantation were consistently low, particularly during vulnerable 6–12 and 6–24 month intervals after PCI. Due to missing comparative data, these findings should be considered exploratory and hypothesis generating. Pairwise meta‐analyses showed no significant differences in safety and efficacy outcomes between BA and DES. Further randomised trials with longer follow‐up are warranted to determine whether the mechanistic profile of BA translates into clinical benefit.

## Author Contributions

Simon Woelbert: conceptualisation, methodology, data curation, formal analysis, investigation, visualisation; writing – original draft, writing – review and editing. Stephanie Kuehne: data curation, risk of bias assessment; writing – review and editing. Andrea Patrignani: formal analysis, methodology, statistical analysis; writing – review and editing. Mauro Chiarito: methodology, formal analysis; writing – review and editing. Jan Torzewski: writing – review and editing, supervision. Philip Raake: supervision, writing – review and editing. Dario Bongiovanni: conceptualisation, methodology, formal analysis, supervision, writing – review and editing.

## Funding

The authors have nothing to report.

## Ethics Statement

No additional ethics approval was required for this systematic review and meta‐analysis, as the study was based exclusively on previously published data from studies that had obtained approval from their respective local ethics committees.

## Conflicts of Interest

The authors declare no conflicts of interest.

## Supporting information


**Figure S1:** Pairwise meta‐analysis of target‐lesion failure components at 1 year.
**Figure S2:** Pooled clinically‐driven target‐lesion revascularisation rates between 6 and 12 months after DynamX bioadaptor implantation.
**Figure S3:** Pooled target‐vessel myocardial infarction rates between 6 and 12 months after DynamX bioadaptor implantation.
**Figure S4:** Pooled rate of cardiac death between 6 and 24 months after DynamX bioadaptor implantation.
**Figure S5:** Pooled target‐lesion revascularisation rates between 6 and 24 months after DynamX bioadaptor implantation.
**Figure S6:** Pooled target‐vessel myocardial infarction rates between 6 and 24 months after DynamX bioadaptor implantation.
**Figure S7:** Pooled incidence of cardiac death at 12 months.
**Figure S8:** Pooled incidence of target‐vessel myocardial infarction at 12 months.
**Figure S9:** Pooled incidence of target‐lesion revascularisation at 12 months.
**Figure S10:** Leave‐one‐out analysis of pooled clinically‐driven target‐lesion revascularisation rates between 6 and 12 months.
**Figure S11:** Leave‐one‐out analysis of pooled target‐lesion failure rates between 6 and 24 months.
**Figure S12:** Leave‐one‐out analysis of pooled target‐lesion revascularisation rates between 6 and 24 months.
**Figure S13:** Sensitivity analysis excluding abstract‐only studies. Forest plots showing the pooled incidence of target‐lesion failure and clinically driven target‐lesion revascularisation between 6 and 12 months after bioadaptor implantation, calculated using a random‐effects generalised linear mixed model (GLMM). Estimates are presented as events per 100 observations with 95% confidence intervals.
**Figure S14:** Study‐level meta‐regression of 12‐month clinically‐driven target‐lesion revascularisation (TLR) according to prevalence of acute coronary syndrome (ACS), diabetes mellitus (DM) and mean age (MA).
**Table S1:** Overview of included studies: design, trial registration, geographic setting and recruitment period.
**Table S2:** Values represent patient‐weighted means or proportions across included randomised controlled trials. Weighting was performed according to the number of patients contributing data for each variable. No statistical comparisons were performed.
**Table S3:** Mean weighted lesion characteristics were calculated by weighting study‐level estimates according to the number of lesions contributing data for each variable, separately for the bioadaptor and drug‐eluting stent groups.
**Table S4:** Mean weighted clinical characteristics were calculated by weighting study‐level estimates by the number of patients contributing data for each variable, across bioadaptor arms from pairwise and single‐arm studies.
**Table S5:** Weighted lesion characteristics were calculated by weighting study‐level estimates according to the number of lesions contributing data for each variable.
**Table S6:** Risk of bias assessment for single‐arm studies using JBI Critical Appraisal Checklist for Case Series.
**Table S7:** Risk of bias assessment for randomised‐controlled trials using RoB2.

## Data Availability

The data that support the findings of this study are available from the corresponding author upon reasonable request.
