# Supplementary material for: A Comprehensive Meta‐Analysis of Bioadaptor Versus Drug‐Eluting Stents in Randomised Trials With Exploratory Single‐Arm Landmark Analyses
Source: Eur J Clin Invest. 2026 May 5;56:e70217. doi: 10.1111/eci.70217 (PMC13145319; doi:10.1111/eci.70217)
Supplement: Supplementary file 1 — Figure S1: Pairwise meta‐analysis of target‐lesion failure components at 1 year. Figure S2: Pooled clinically‐driven target‐lesion revascularisation rates between 6 and 12 months after DynamX bioadaptor implantation. Figure S3: Pooled target‐vessel myocardial infarction rates between 6 and 12 months after DynamX bioadaptor implantation. Figure S4: Pooled rate of cardiac death between 6 and 24 months after DynamX bioadaptor implantation. Figure S5: Pooled target‐lesion revascularisation rates between 6 and 24 months after DynamX bioadaptor implantation. Figure S6: Pooled target‐vessel myocardial infarction rates between 6 and 24 months after DynamX bioadaptor implantation. Figure S7: Pooled incidence of cardiac death at 12 months. Figure S8: Pooled incidence of target‐vessel myocardial infarction at 12 months. Figure S9: Pooled incidence of target‐lesion revascularisation at 12 months. Figure S10: Leave‐one‐out analysis of pooled clinically‐driven target‐lesion revascularisation rates between 6 and 12 months. Figure S11: Leave‐one‐out analysis of pooled target‐lesion failure rates between 6 and 24 months. Figure S12: Leave‐one‐out analysis of pooled target‐lesion revascularisation rates between 6 and 24 months. Figure S13: Sensitivity analysis excluding abstract‐only studies. Forest plots showing the pooled incidence of target‐lesion failure and clinically driven target‐lesion revascularisation between 6 and 12 months after bioadaptor implantation, calculated using a random‐effects generalised linear mixed model (GLMM). Estimates are presented as events per 100 observations with 95% confidence intervals. Figure S14: Study‐level meta‐regression of 12‐month clinically‐driven target‐lesion revascularisation (TLR) according to prevalence of acute coronary syndrome (ACS), diabetes mellitus (DM) and mean age (MA). Table S1: Overview of included studies: design, trial registration, geographic setting and recruitment period. Table S2: Values represent patient‐weighted means [file ECI-56-e70217-s001.docx]

**A comprehensive meta-analysis of bioadaptor versus drug-eluting stents in randomized trials with exploratory single-arm landmark analyses**

Simon Wölbert^1^ MD; Stephanie Kühne^1^ MD; Andrea Patrignani^2^ MSc; Mauro Chiarito^2^ MD, PhD; Moritz von Scheidt^3,4^ MD; Jan Torzewski^5^ MD; Philip Raake^1^ MD; Dario Bongiovanni^1^ MD, PhD

^1^ Department of Internal Medicine I, Cardiology, University Hospital Augsburg, University of Augsburg, Germany

^2^ Department of Biomedical Sciences, Humanitas University, Pieve Emanuele, Milan, Italy; Humanitas Research Hospital IRCCS, Rozzano, Milano, Italy

^3^ Department of Cardiology, German Heart Center, TUM University Hospital, Technical University Munich, Munich, Germany

^4^ German Center for Cardiovascular Research (DZHK), Partner Site Munich Heart Alliance, Munich, Germany

^5^ Cardiovascular Center Oberallgäu-Kempten, Kempten, Germany

Running title: Bioadaptor Meta-Analysis

**Address for correspondence:**

Professor Dario Bongiovanni, MD, PhD

Department of Internal Medicine I, Cardiology

University Hospital Augsburg

University of Augsburg

Stenglinstraße 2, 86156 Augsburg, Germany

Phone: + 49 0821-400-2355

E-mail: dario.bongiovanni@med.uni-augsburg.de

Table of contents

[Supplementary figures 3](#_Toc226626885)

[Supplementary tables 20](#_Toc226626886)

[Supplementary appendix 26](#_Toc226626887)

[PubMed 29](#_Toc226626888)

[Embase via ovid 29](#_Toc226626889)

[CENTRAL 29](#_Toc226626890)

[Google Scholar 29](#_Toc226626891)

**Supplementary material**

# Supplementary figures

| 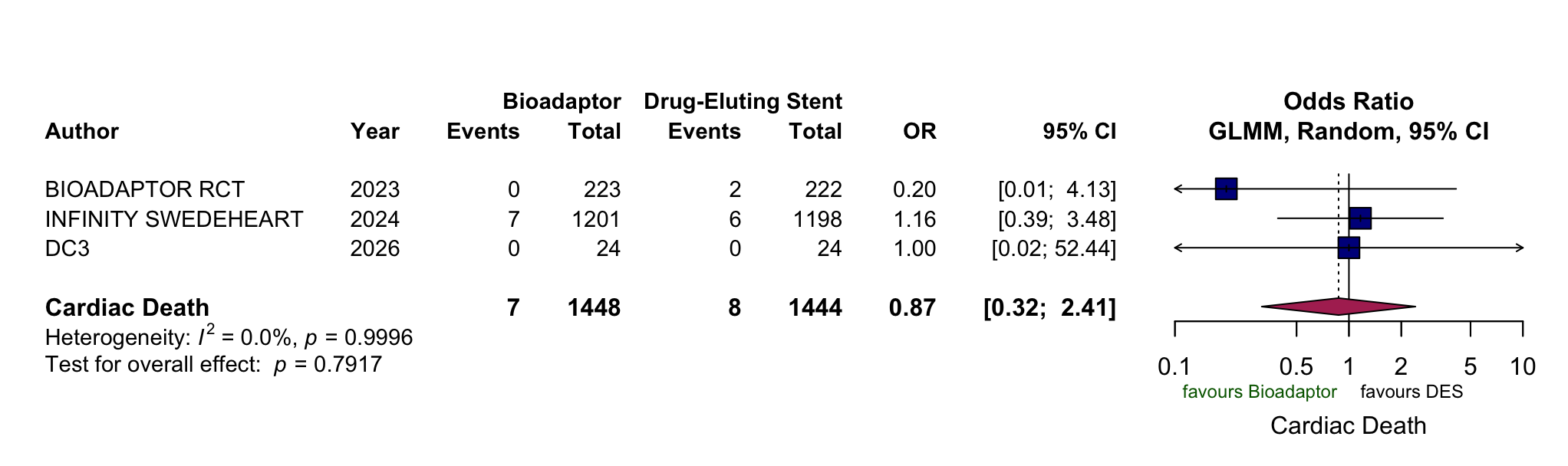  **A** |
| --- |
| 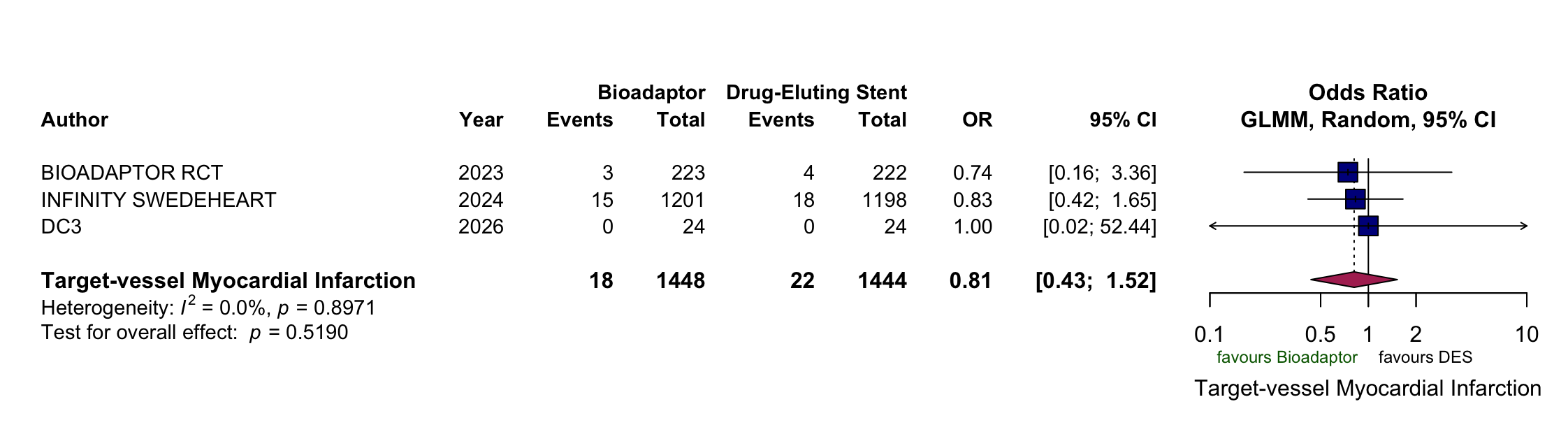  **B** |
| 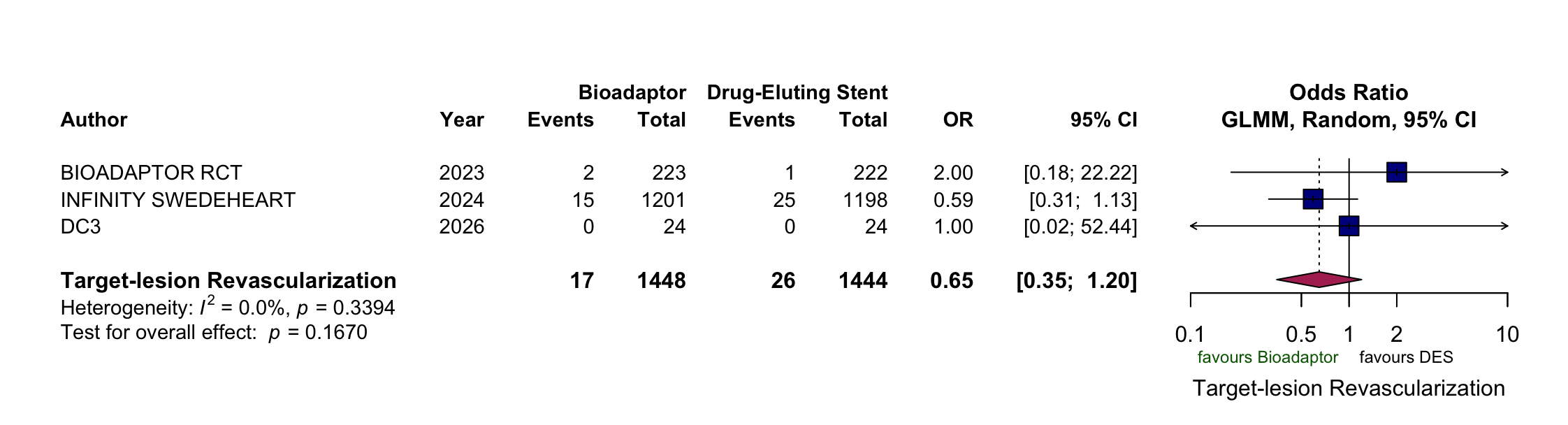  **C** |
| 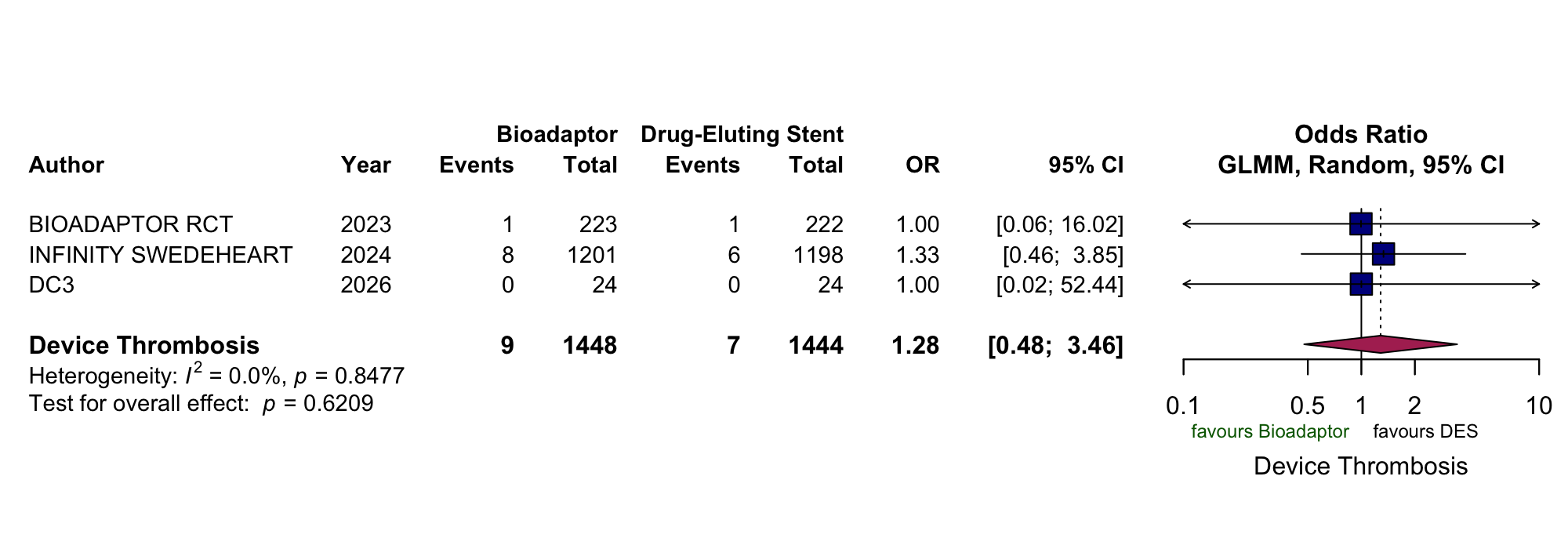  **D** |

Supplementary figure 1: Pairwise meta-analysis of target-lesion failure components at 1 year

Odds ratios (OR) with 95% confidence intervals (CI) for target-lesion failure comparing bioadaptor with drug-eluting stents (DES) were calculated. A random-effects generalized linear mixed effects model (GLMM) was applied to estimate pooled effects. Heterogeneity was assessed using the I² statistic. **A:** cardiac death; **B:** target-vessel myocardial infarction; **C:** target-lesion revascularization; **D:** device thrombosis


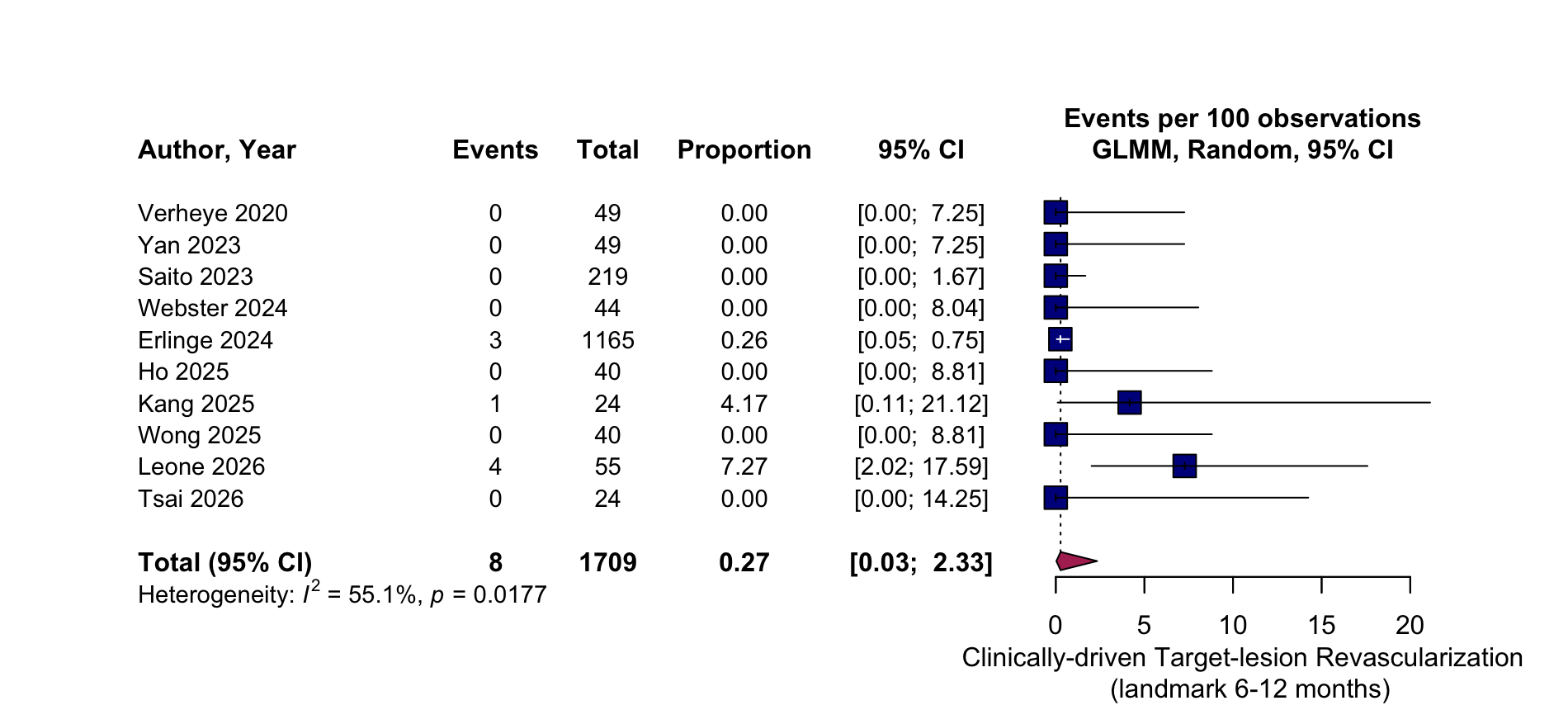


Supplementary figure 2: Pooled clinically-driven target-lesion revascularization rates between 6 and 12 months after DynamX bioadaptor implantation

Event rates are shown per 100 observations and were pooled using a generalized linear mixed model (GLMM) with random effects. Horizontal lines denote 95% confidence intervals (CI). Between-study heterogeneity is reported as I².


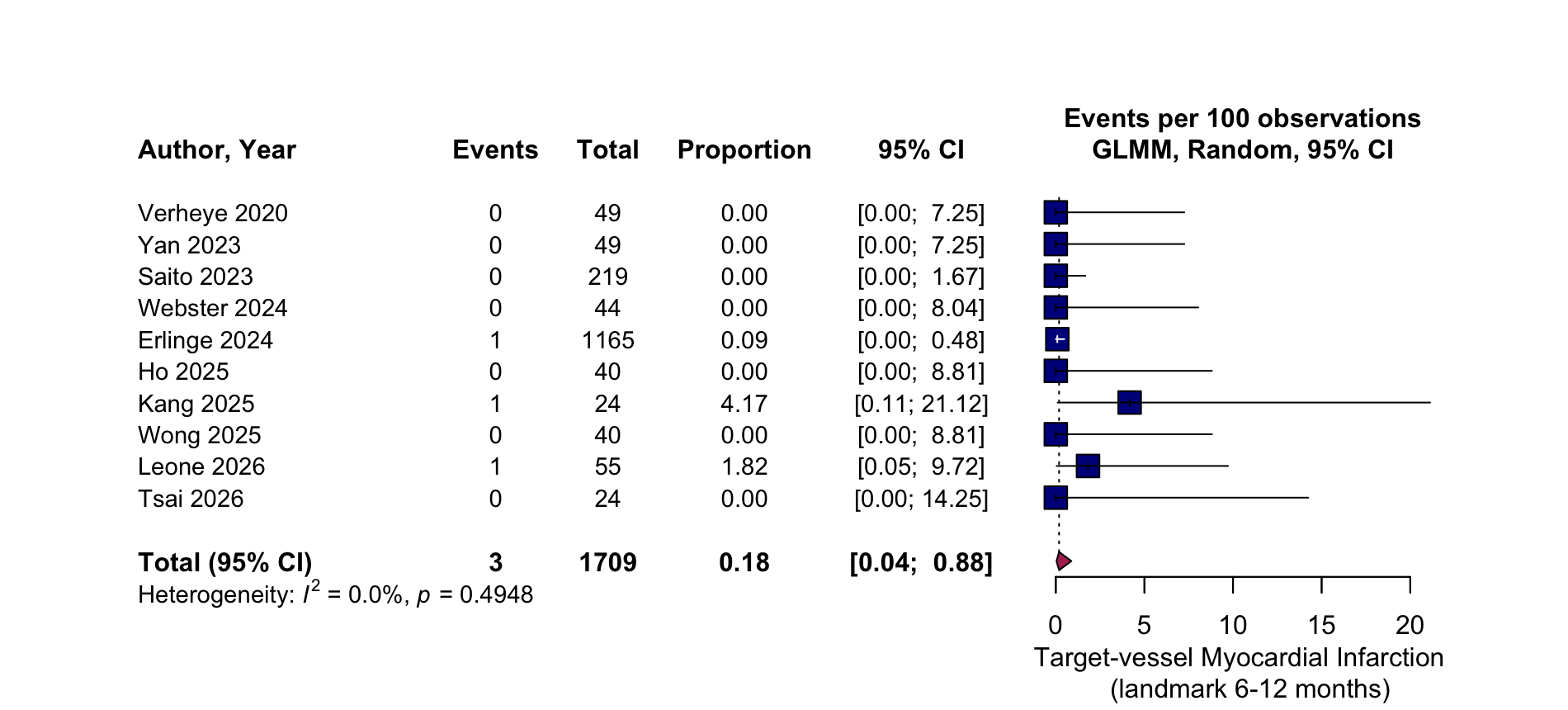


Supplementary figure 3: Pooled target-vessel myocardial infarction rates between 6 and 12 months after DynamX bioadaptor implantation

Event rates are shown per 100 observations and were pooled using a generalized linear mixed model (GLMM) with random effects. Horizontal lines denote 95% confidence intervals (CI). Between-study heterogeneity is reported as I².


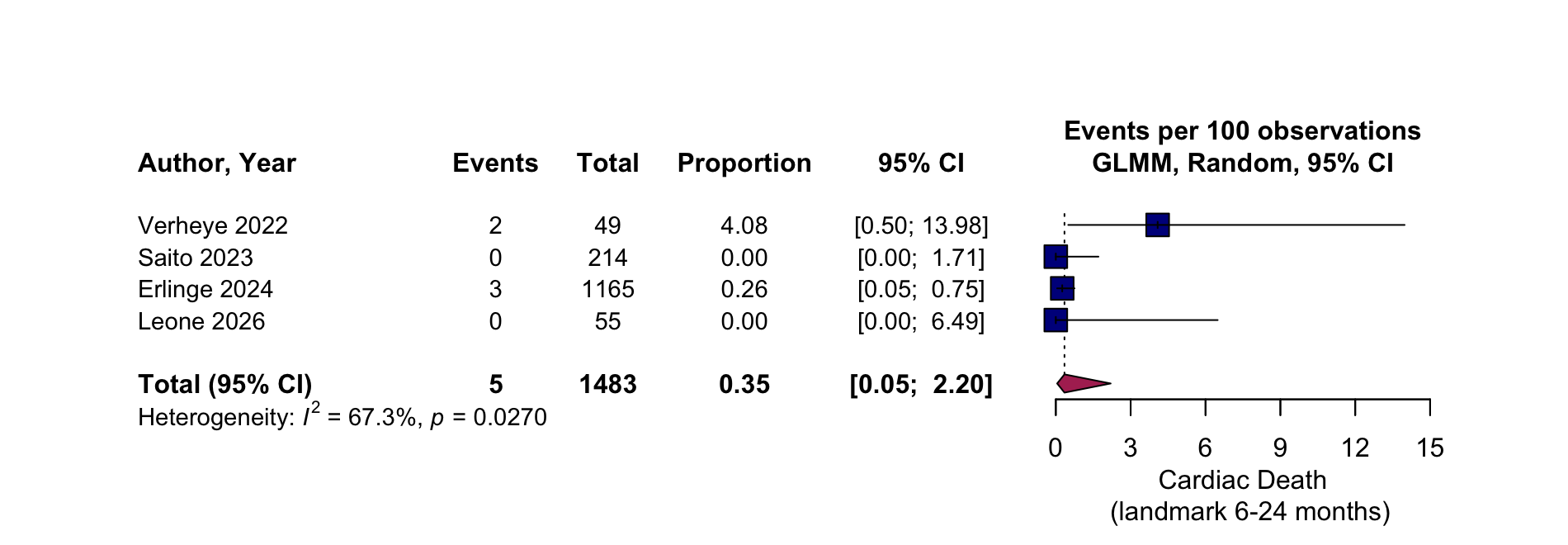


Supplementary figure 4: Pooled rate of cardiac death between 6 and 24 months after DynamX bioadaptor implantation

Event rates are shown per 100 observations and were pooled using a generalized linear mixed model (GLMM) with random effects. Horizontal lines denote 95% confidence intervals (CI). Between-study heterogeneity is reported as I².


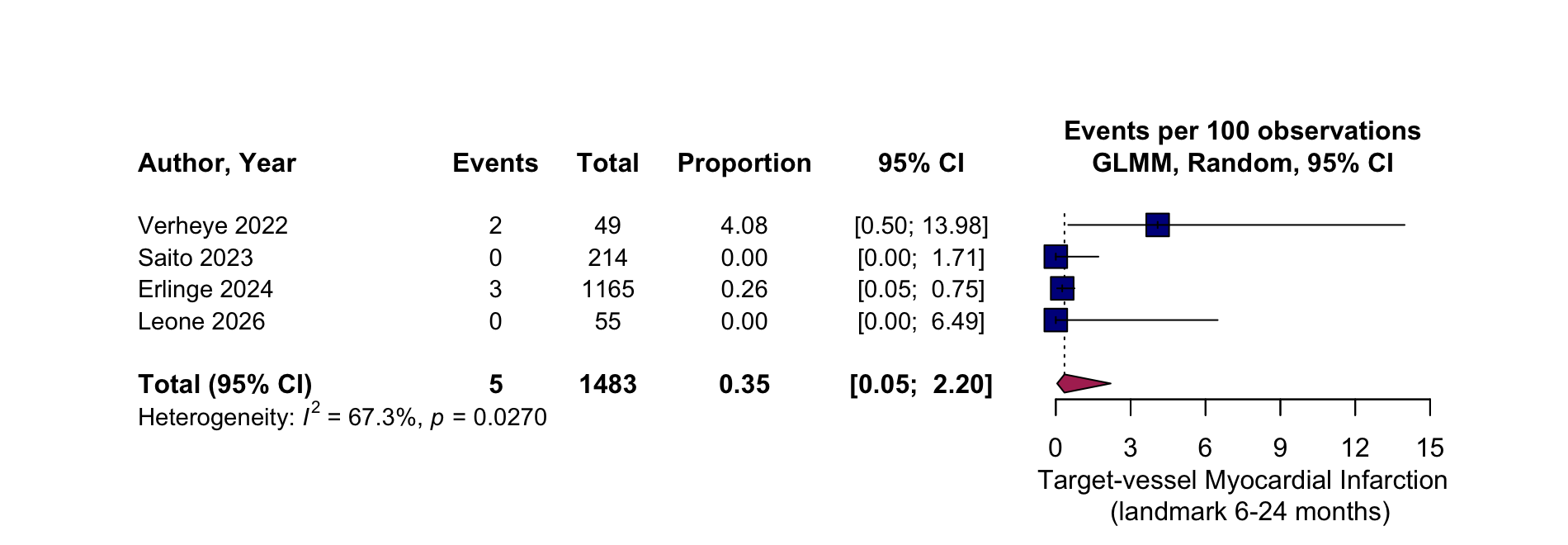


Supplementary figure 5: Pooled target-lesion revascularization rates between 6 and 24 months after DynamX bioadaptor implantation

Event rates are shown per 100 observations and were pooled using a generalized linear mixed model (GLMM) with random effects. Horizontal lines denote 95% confidence intervals (CI). Between-study heterogeneity is reported as I².


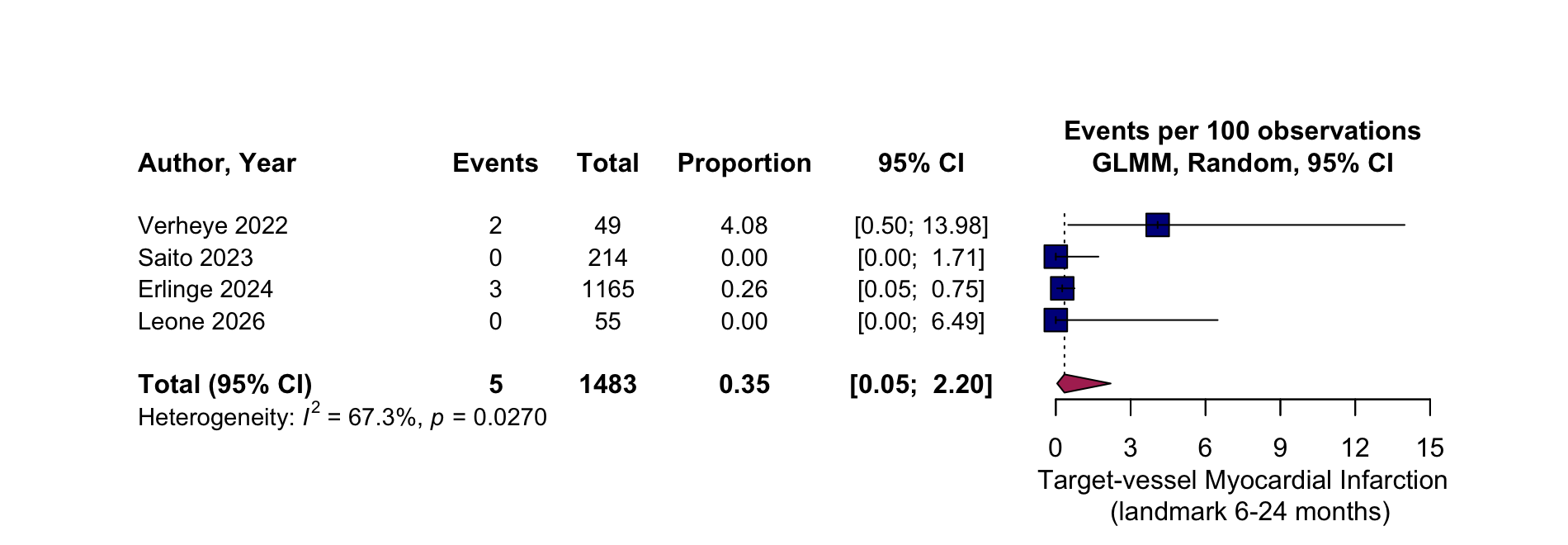


Supplementary figure 6: Pooled target-vessel myocardial infarction rates between 6 and 24 months after DynamX bioadaptor implantation

Event rates are shown per 100 observations and were pooled using a generalized linear mixed model (GLMM) with random effects. Horizontal lines denote 95% confidence intervals (CI). Between-study heterogeneity is reported as I².


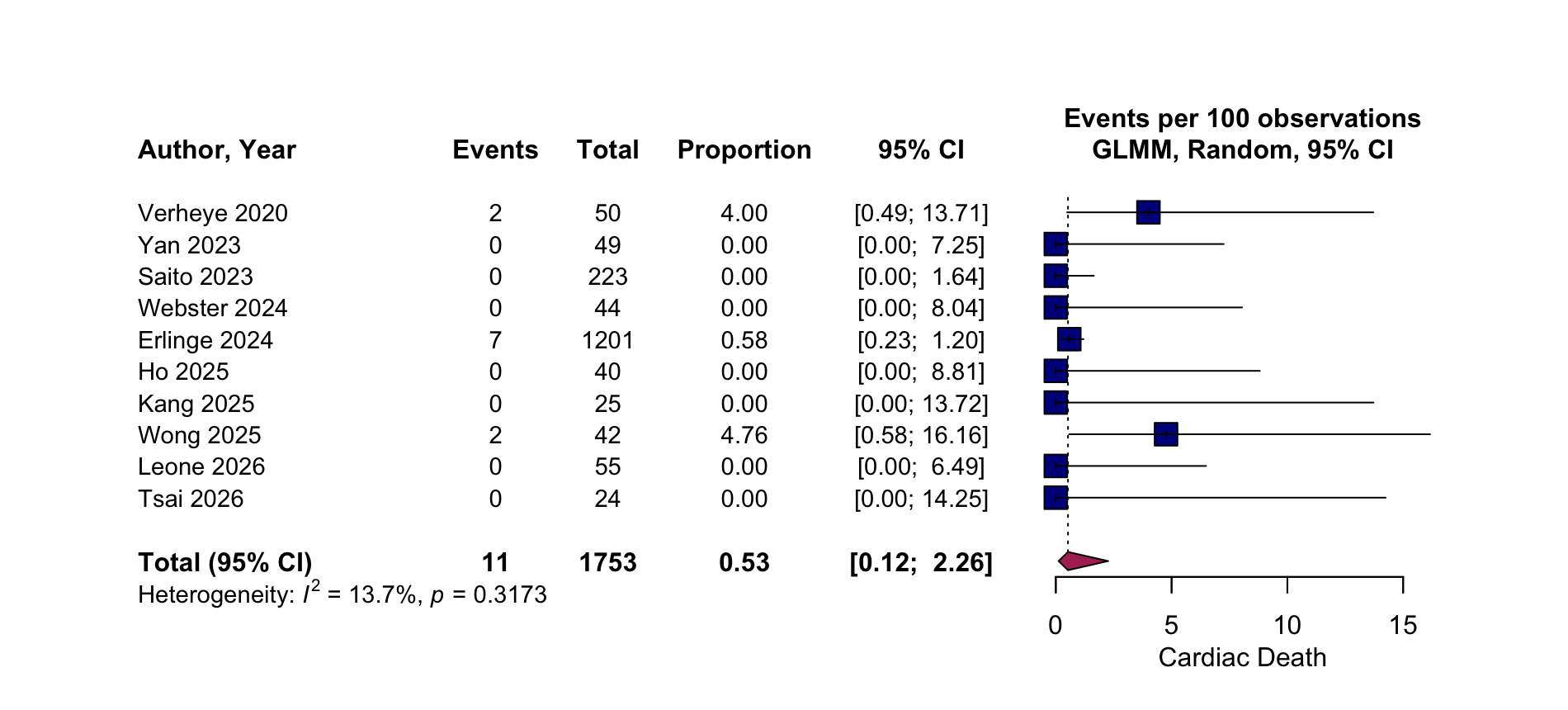


Supplementary figure 7: Pooled incidence of cardiac death at 12 months

Event rates are shown per 100 observations and were pooled using a generalized linear mixed model (GLMM) with random effects. Horizontal lines denote 95% confidence intervals (CI). Between-study heterogeneity is reported as I².


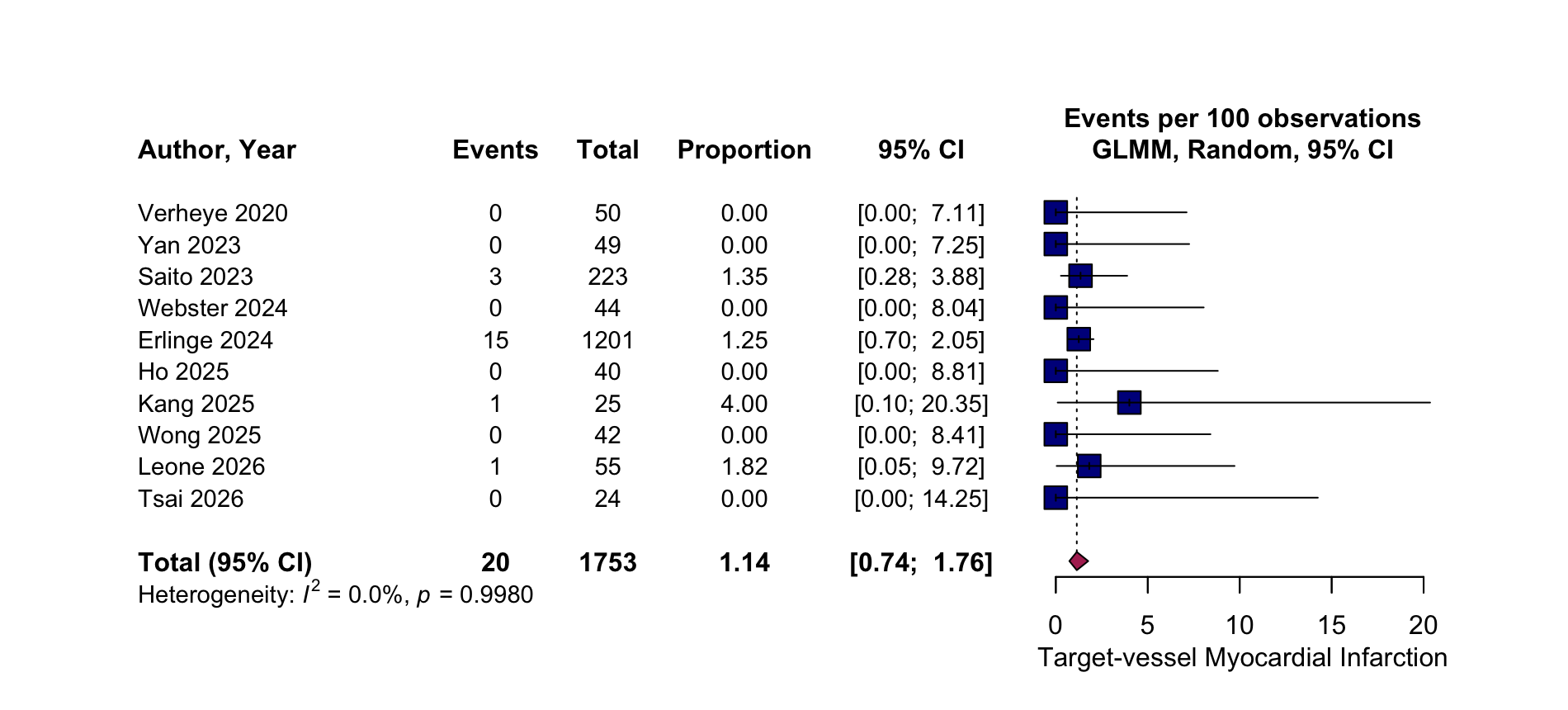


Supplementary figure 8: Pooled incidence of target-vessel myocardial infarction at 12 months

Event rates are shown per 100 observations and were pooled using a generalized linear mixed model (GLMM) with random effects. Horizontal lines denote 95% confidence intervals (CI). Between-study heterogeneity is reported as I².


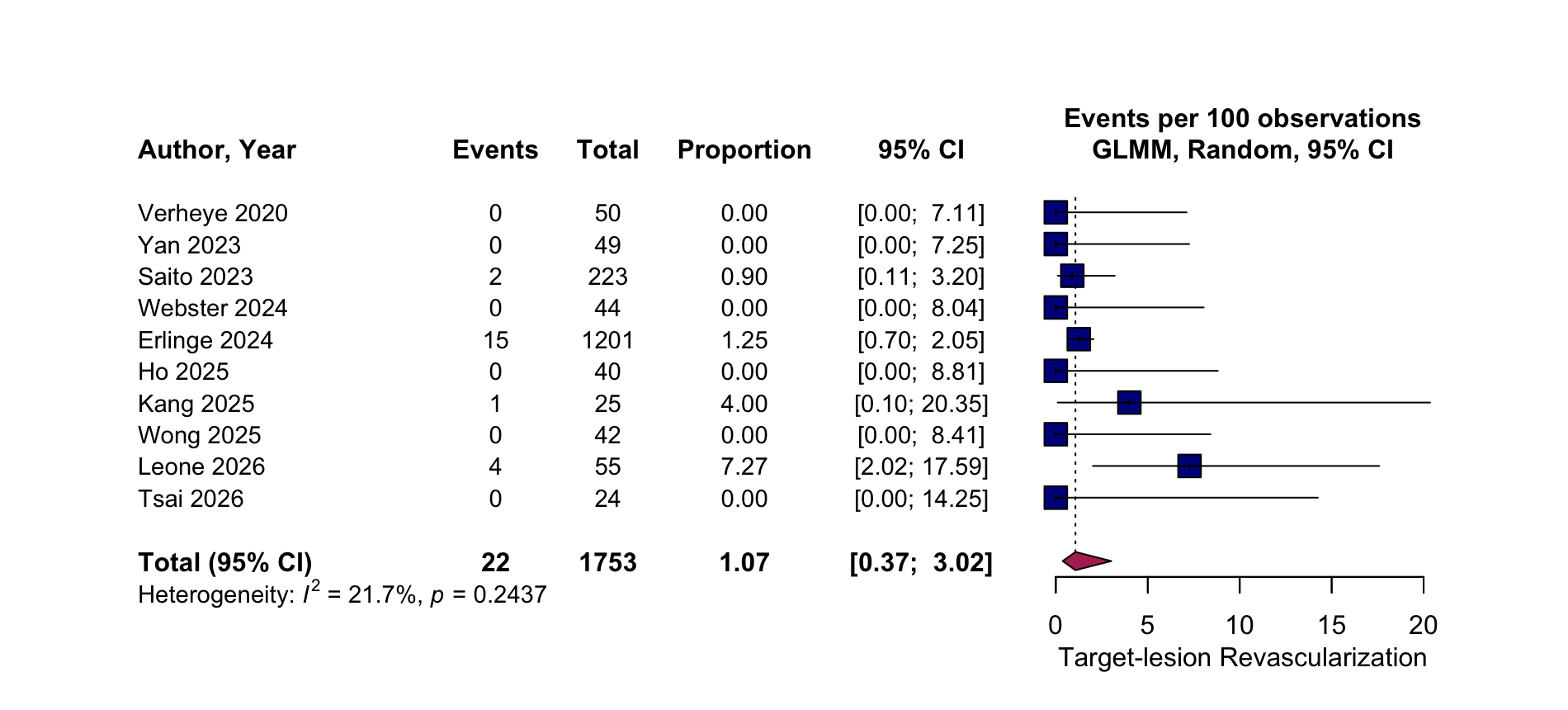


Supplementary figure 9: Pooled incidence of target-lesion revascularization at 12 months

Event rates are shown per 100 observations and were pooled using a generalized linear mixed model (GLMM) with random effects. Horizontal lines denote 95% confidence intervals (CI), and the diamond indicates the pooled estimate. Between-study heterogeneity is reported as I².


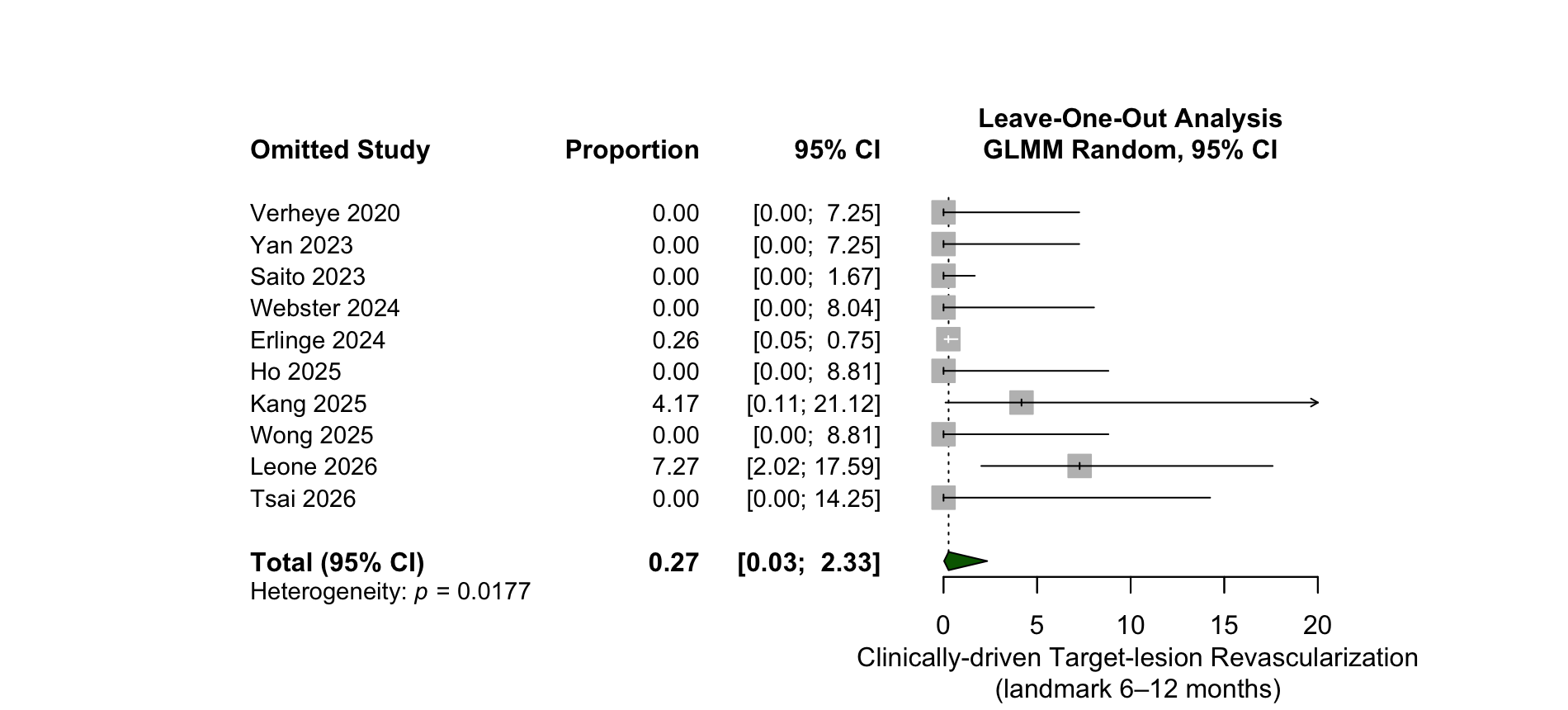


Supplementary figure 10: Leave-one-out analysis of pooled clinically-driven target-lesion revascularization rates between 6 and 12 months

Sequential omission of individual studies demonstrated that heterogeneity was largely driven by **Leone 2026 and Erlinge 2024,** with complete resolution of heterogeneity when these studies were excluded (I² = 0.0%), while the pooled TLR estimate remained largely unchanged. CI: confidence interval; GLMM: generalized linear mixed model with random effects. Residual I^2^ (sequential order): 60.1%; 60.1%; 60.1%; 60.1%; 0.0%; 60.1%; 58.6%; 60.1%; 0.0%; 60.1%.


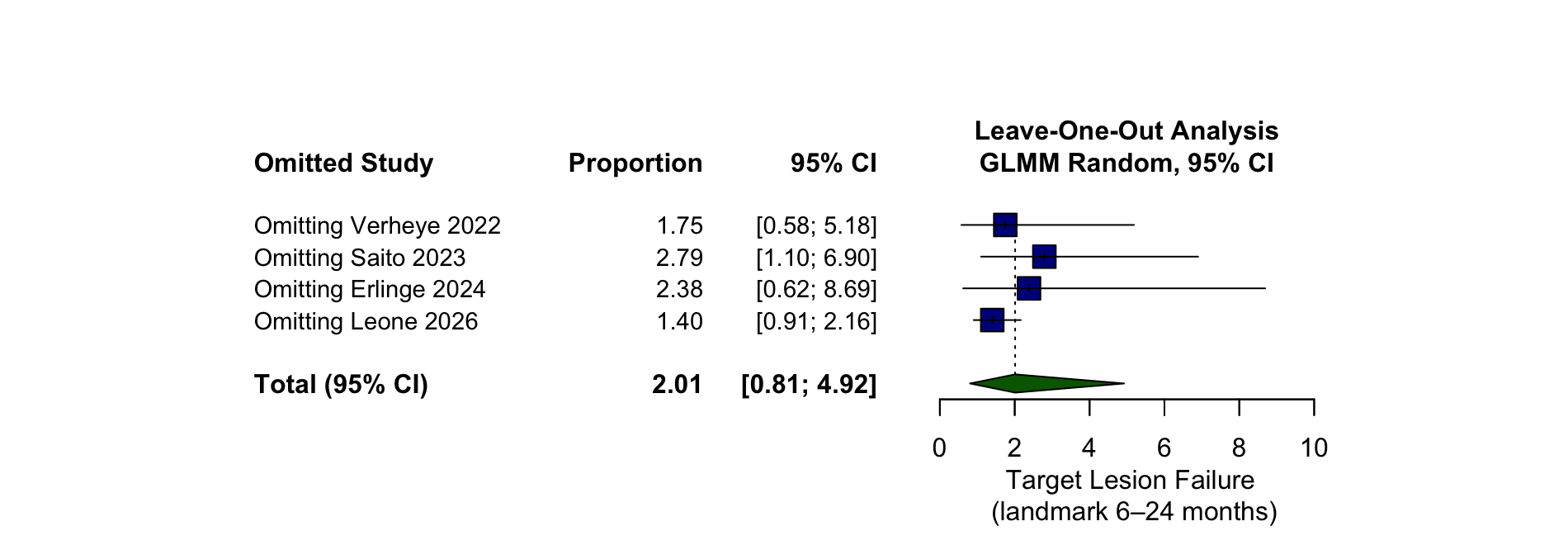


Supplementary figure 11: Leave-one-out analysis of pooled target-lesion failure rates between 6 and 24 months

TLF incidence remained robust across study exclusions, with point estimates ranging from 1.40% to 2.84%. Overall heterogeneity was high in the primary analysis (I² ≈ 75%) but was substantially reduced when individual studies were omitted, most notably after exclusion of Leone et al. 2026 (I² reduced to 41.4%). Residual I^2^ (sequential order): 81.6%; 79.6%; 68.7%; 41.4%.


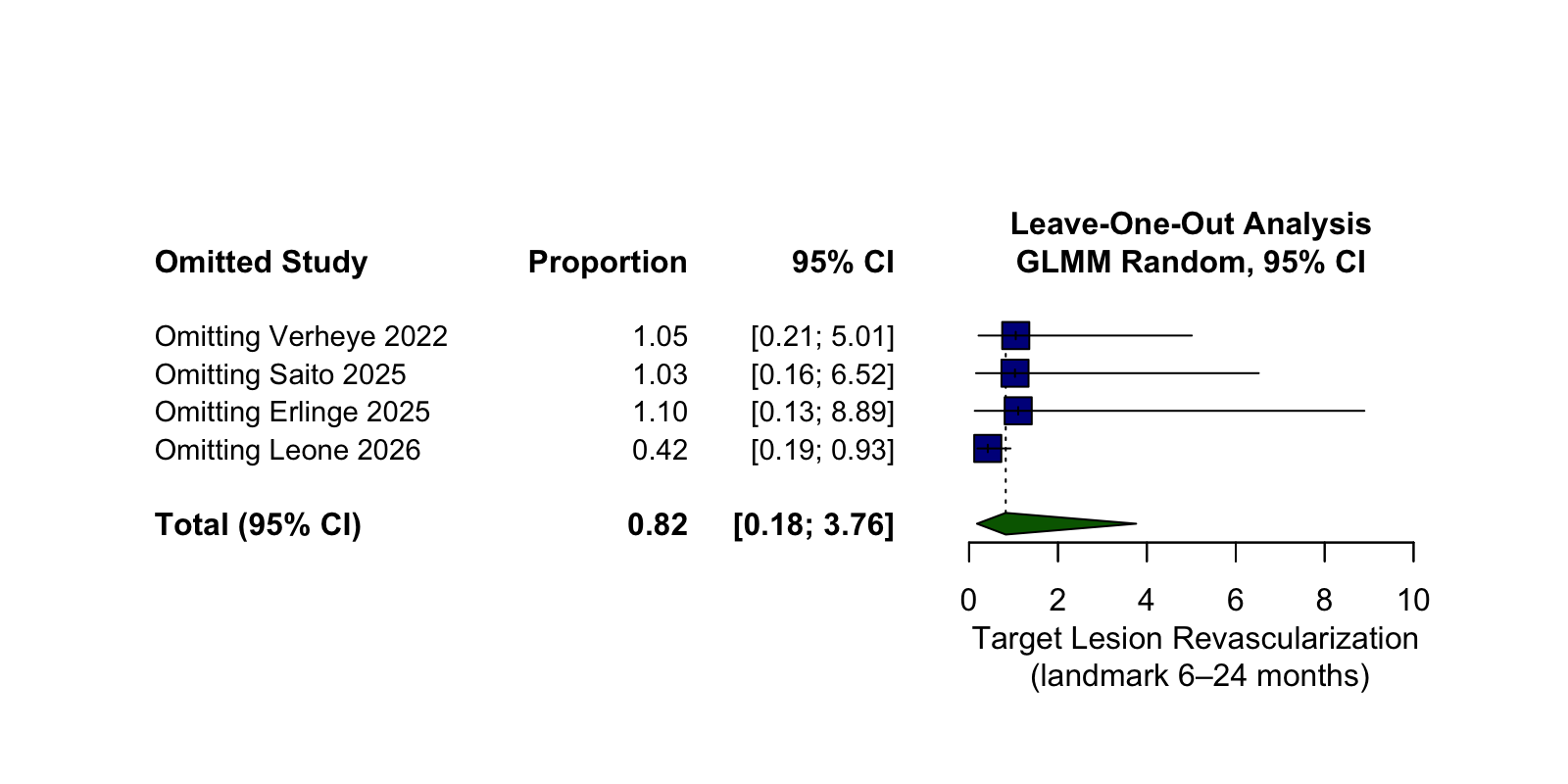


Supplementary figure 12: Leave-one-out analysis of pooled target-lesion revascularization rates between 6 and 24 months

Pooled TLR estimates remained largely stable when Verheye et al., Saito et al., or Erlinge et al. were excluded (range 1.03%-1.10%), whereas exclusion of Leone et al. reduced the estimate to 0.42%. Overall heterogeneity was high in the primary analysis (I² ≈ 84%) but dropped to 0% when Leone et al. was excluded. Residual I² values in sequential order were 89.5%, 88.8%, 67.9%, and 0.0%.

| 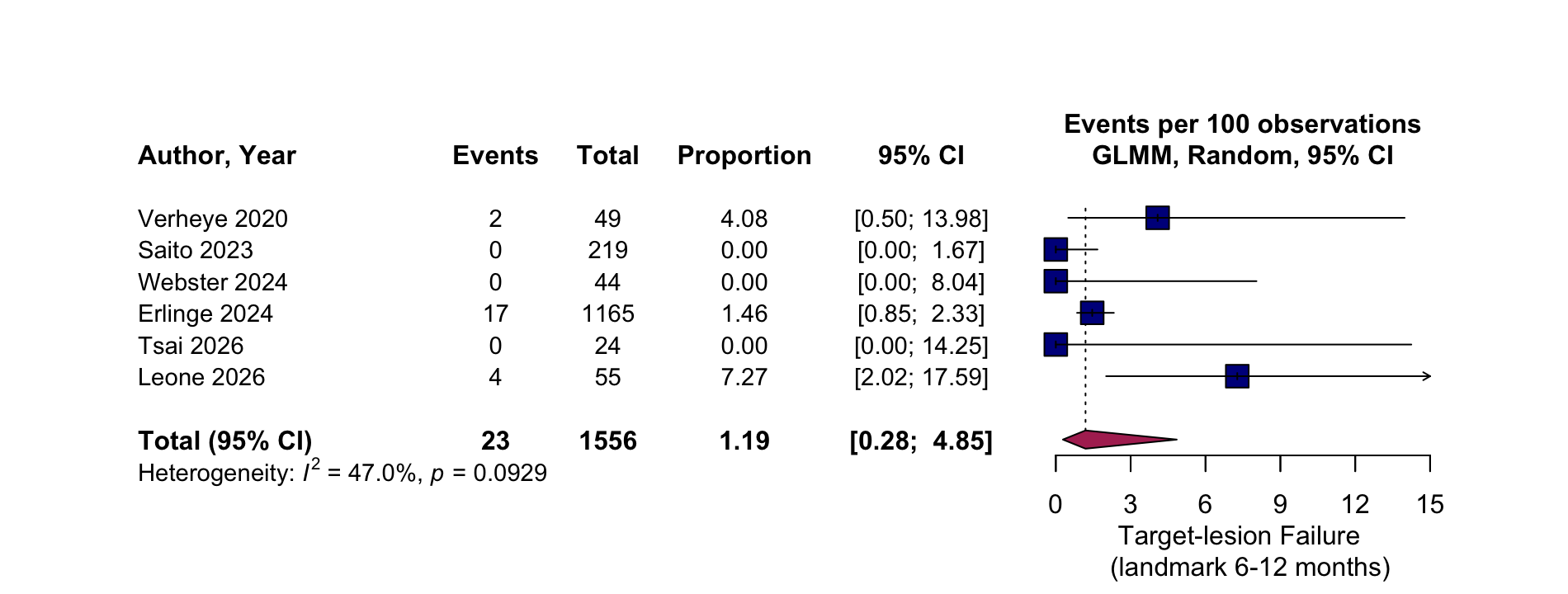  **A** |
| --- |
| 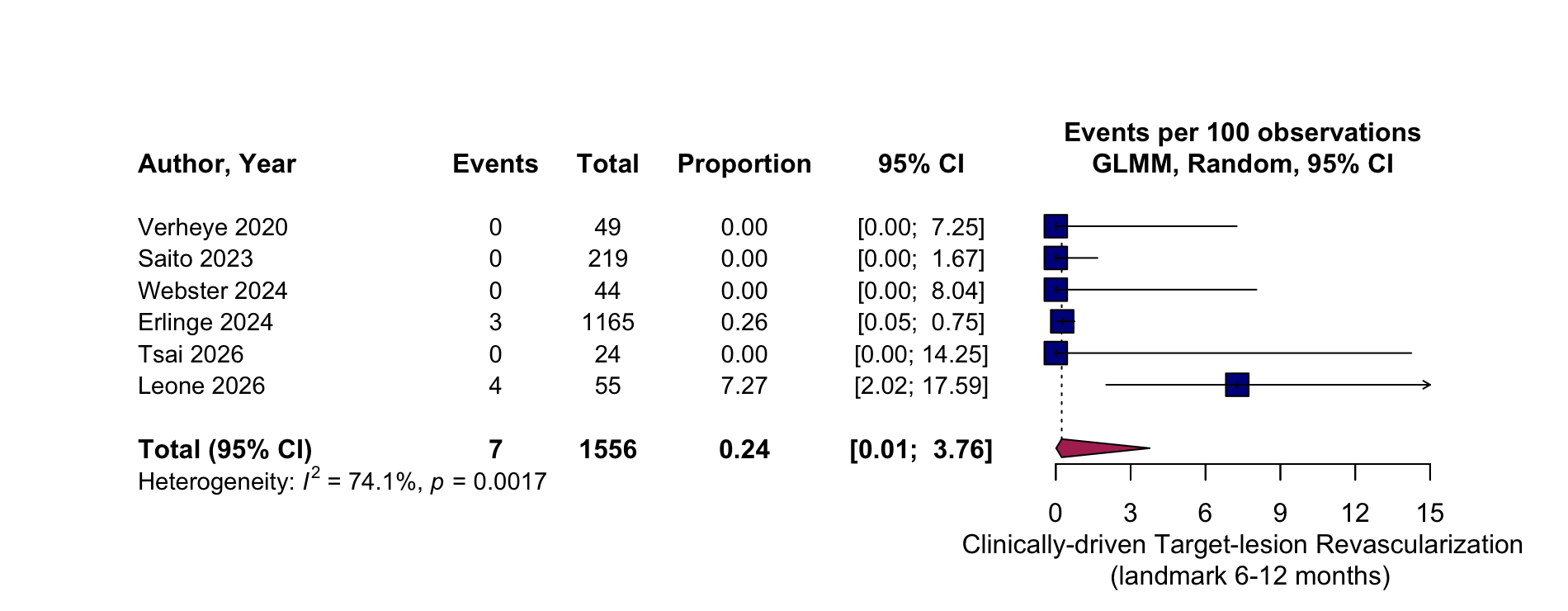  **B** |

Supplementary figure 13: **Sensitivity analysis excluding abstract-only studies.** Forest plots showing the pooled incidence of target-lesion failure and clinically driven target-lesion revascularization between 6 and 12 months after bioadaptor implantation, calculated using a random-effects generalized linear mixed model (GLMM). Estimates are presented as events per 100 observations with 95% confidence intervals.


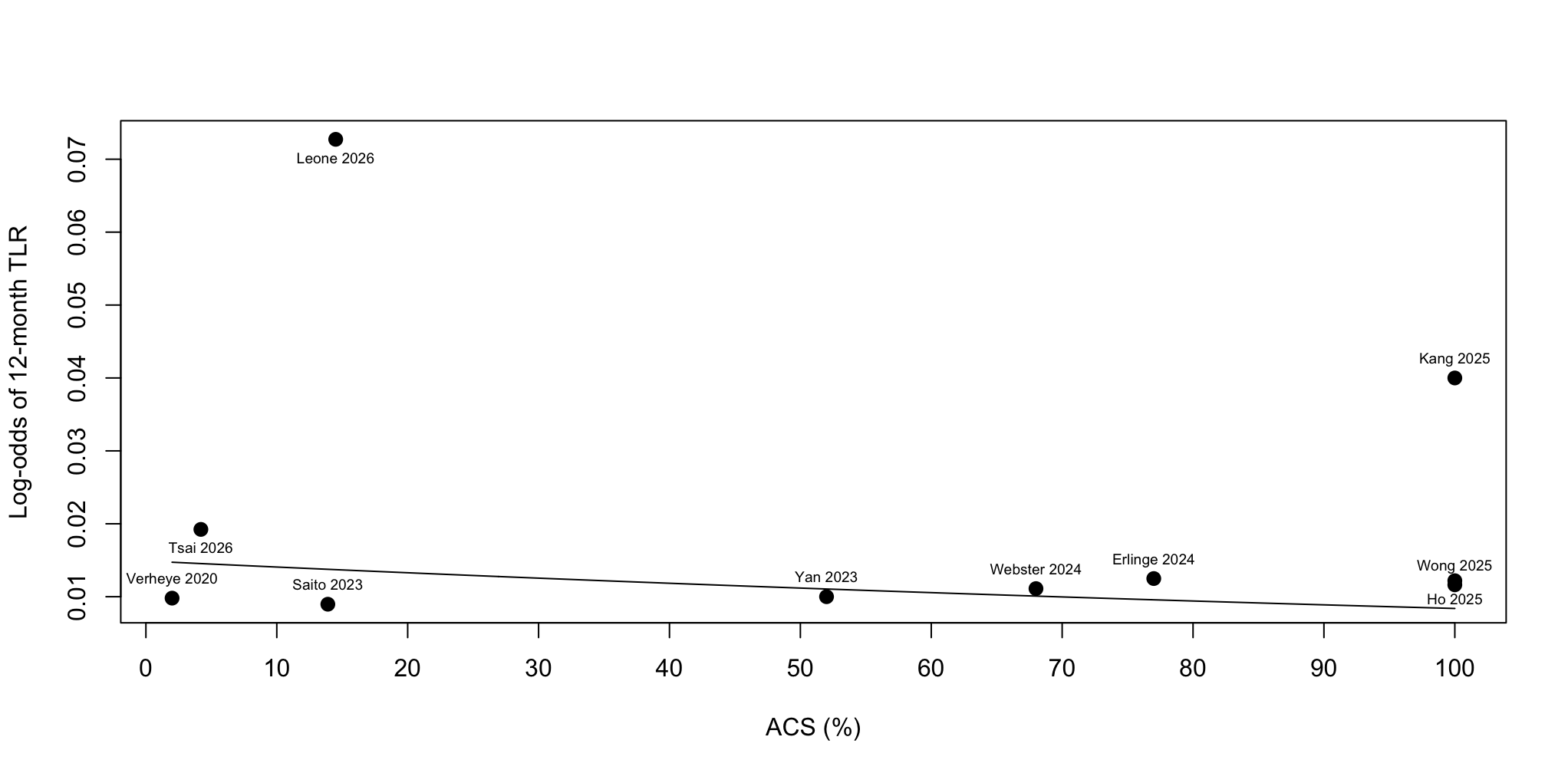

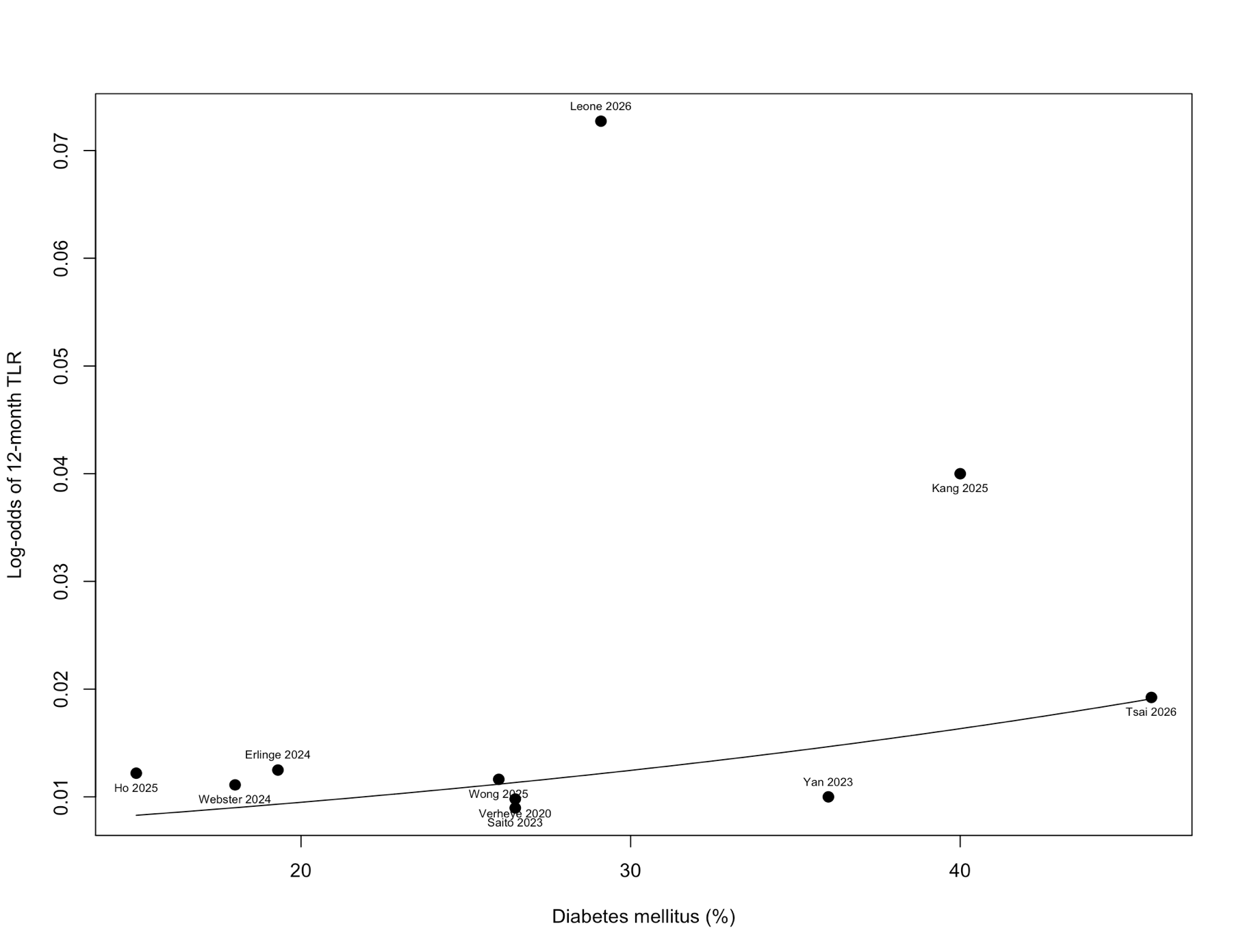

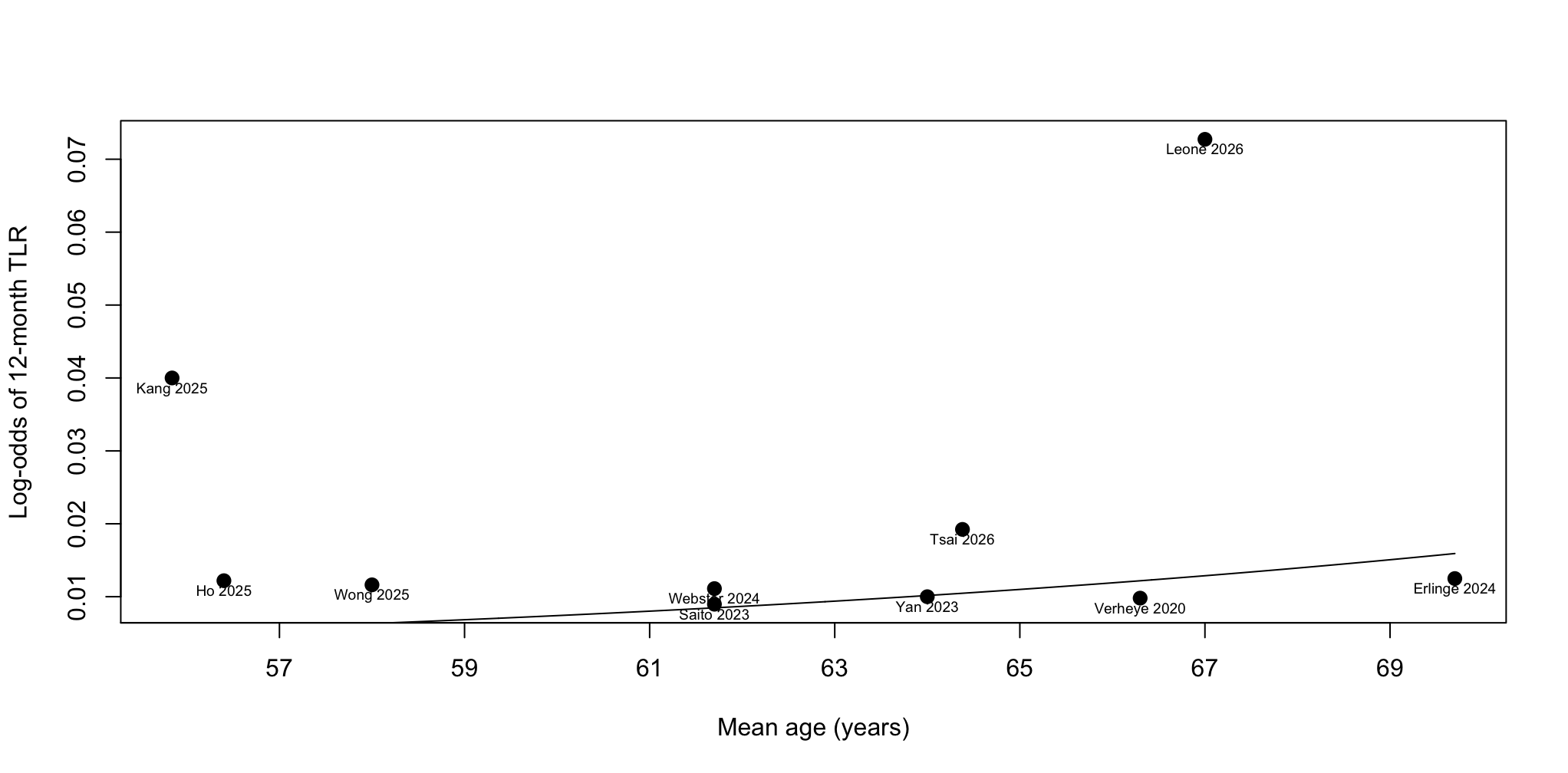


A

B

C

Supplementary figure 14: Study-level meta-regression of 12-month clinically-driven target-lesion revascularization (TLR) according to prevalence of acute coronary syndrome (ACS), diabetes mellitus (DM), and mean age (MA)

None of the clinical study-level covariates showed a significant association with 12-month clinically-driven TLR. **(A)** ACS presentation (per 10% increase; OR 0.93, 95% CI 0.75-1.14; p=0.470); **(B)** DM (per 10% increase; OR 1.74, 95% CI 0.60-5.09; p=0.312); **(C)** MA (per 10-year increase; OR 2.18, 95% CI 0.31-15.32; p=0.434), did not explain the between-study variability in TLR rates. The wide confidence intervals reflect the very low number of TLR events across studies, indicating limited statistical power and supporting an exploratory interpretation of these findings.

# Supplementary tables

| Study | Design | NCT identifier | Study location | Recruitment period | Type of publication |
| --- | --- | --- | --- | --- | --- |
| Verheye, 2020^1^ | POR | 03429894 | 6 hospitals in Belgium and Italy | Nov 2017 to Sept 2018 | Peer-reviewed journal |
| Yan, 2023^2^ | POR | 04483791 | 4 tertiary hospitals in Hong Kong | Oct 2020 to Oct 2021 | Conference abstract |
| Saito, 2023^3^ | RCT | 04192747 | 34 hospitals in Europe, Japan and New Zealand | Jan 2021 to Feb 2022 | Peer-reviewed journal |
| Webster, 2024^4^ | POR | 03634020 | 7 hospitals in New Zealand | Exact period NA | Peer-reviewed journal |
| Erlinge, 2024^5^ | RCT | 04562805 | 20 hospitals in Sweden | Sept 2020 to July 2023 | Peer-reviewed journal |
| Leone, 2026^6^ | POR | 05464147 | Rozzano, Italy, Humanitas Research Hospital | Aug 2021 to Sept 2023 | Peer-reviewed journal |
| Ho, 2025^7^ | POR | NA | Tan Tock Seng Hospital, Singapore | 2021 to 2022 | Conference abstract |
| Kang, 2025^8^ | ROR | NA | Hospital Sultanah Bahiyah, Malaysia | Jan 2021 to Jan 2024 | Conference abstract |
| Wong, 2025^9^ | ROR | NA | United Christian Hospital in Hong Kong | Apr 2022 to Oct 2023 | Conference abstract |
| Tsai, 2026^10^ | RCT | NCT04936191 | National Taiwan University Hospital | Nov 2023 to Dec 2024 | Peer-reviewed journal |

Supplementary table S1: Overview of included studies: design, trial registration, geographic setting, and recruitment period

NA: not available; NCT: National Clinical Trial; RCT: randomized controlled trial; POR: prospective observational registry; ROR: retrospective observational registry

| **Variable** | **Bioadaptor**  **(n=1448)** | **Drug-eluting stent**  **(n=1444)** | **Weighting method** |
| --- | --- | --- | --- |
| Age, years | 69.2 | 68.7 | Patient-weighted mean |
| Male sex, % | 76.4 | 76.7 | Patient-weighted proportion |
| ACS at presentation, % | 66.0 | 64.8 | Patient-weighted proportion |
| Smoking history, % | 22.5 | 24.0 | Patient-weighted proportion |
| Diabetes mellitus, % | 20.9 | 19.6 | Patient-weighted proportion |
| Hypertension, % | 62.5 | 61.3 | Patient-weighted proportion |
| Dyslipidemia, % | 51.8 | 48.4 | Patient-weighted proportion |
| Previous PCI, % | 18.8 | 18.1 | Patient-weighted proportion |
| Previous CABG, % | 1.0 | 0.7 | Patient-weighted proportion |
| Multivessel disease, % | 12.1 | 13.4 | Patient-weighted proportion |

Supplementary table S2: Values represent patient-weighted means or proportions across included randomized controlled trials. Weighting was performed according to the number of patients contributing data for each variable. No statistical comparisons were performed.

| **Variable** | **Bioadaptor**  **(n=1669 lesions)** | **Drug-eluting stent**  **(n=1685 lesions)** | **Weighting method** |
| --- | --- | --- | --- |
| Lesion length, mm | 23.2 | 23.5 | Lesion-weighted mean |
| Type B2/C lesions, % | 40.1 | 38.7 | Lesion-weighted proportion |
| Diameter stenosis, % | 83.3 | 83.6 | Lesion-weighted mean |
| Reference vessel diameter, mm | 3.18 | 3.11 | Lesion-weighted mean |
| Target vessel LAD, % | 51.0 | 51.0 | Lesion-weighted proportion |
| Target vessel RCA, % | 25.7 | 26.7 | Lesion-weighted proportion |
| Target vessel LCX, % | 23.3 | 22.3 | Lesion-weighted proportion |
| Bifurcation lesions, % | 13.4 | 13.6 | Lesion-weighted proportion |
| Moderate/severe calcification, % | 17.4 | 16.0 | Lesion-weighted proportion |
| Moderate/severe tortuosity, % | 11.0 | 9.7 | Lesion-weighted proportion |
| Predilatation performed, % | 99.8 | 99.9 | Lesion-weighted proportion |
| Postdilatation performed, % | 61.4 | 56.6 | Lesion-weighted proportion |

Supplementary table S3: Mean weighted lesion characteristics were calculated by weighting study-level estimates according to the number of lesions contributing data for each variable, separately for the bioadaptor and drug-eluting stent groups.

| **Variable** | **Bioadaptor (weighted)** | **Weighting method** | **n included** |
| --- | --- | --- | --- |
| Age, years | 68.9 | Patient-weighted mean | 1753 |
| Male sex, % | 77.6 | Patient-weighted proportion | 1753 |
| ACS at presentation, % | 64.9 | Patient-weighted proportion | 1753 |
| Smoking history, % | 31.2 | Patient-weighted proportion | 1688 |
| Diabetes mellitus, % | 22.8 | Patient-weighted proportion | 1753 |
| Hypertension, % | 61.8 | Patient-weighted proportion | 1688 |
| Prior myocardial infarction, % | 23.6 | Patient-weighted proportion | 1435 |
| 12-month follow-up completed, % | 98.9 | Patient-weighted proportion | 1693 |

Supplementary table S4: Mean weighted clinical characteristics were calculated by weighting study-level estimates by the number of patients contributing data for each variable, across bioadaptor arms from pairwise and single-arm studies.

| **Variable** | **Bioadaptor (weighted)** | **Weighting method** | **n included** |
| --- | --- | --- | --- |
| Type B2/C lesions, % | 40.5 | Lesion-weighted proportion | 1875 |
| Lesion length, mm | 22.6 | Lesion-weighted mean | 1820 |
| Target vessel LAD, % | 51.1 | Lesion-weighted proportion | 1900 |

Supplementary table S5: Weighted lesion characteristics were calculated by weighting study-level estimates according to the number of lesions contributing data for each variable.

| Study | Q1 | Q2 | Q3 | Q4 | Q5 | Q6 | Q7 | Q8 | Q9 | Q10 | Overall Risk of Bias |
| --- | --- | --- | --- | --- | --- | --- | --- | --- | --- | --- | --- |
| Verheye, 2020^1^ | YES | YES | YES | YES | YES | YES | YES | YES | YES | YES | Low Risk |
| Yan,  2023^2^ | YES | YES | YES | YES | YES | YES | YES | YES | YES | YES | Low Risk |
| Webster, 2024^4^ | YES | YES | YES | YES | YES | YES | YES | YES | YES | YES | Low Risk |
| Leone, 2026^6^ | YES | YES | YES | YES | YES | YES | YES | YES | YES | YES | Low Risk |
| Ho,  2025^7^ | YES | YES | YES | UNCLEAR | UNCLEAR | YES | YES | YES | YES | YES | Some Concerns |
| Kang, 2025^8^ | YES | UNCLEAR | UNCLEAR | YES | YES | YES | YES | YES | YES | UNCLEAR | High Risk |
| Wong, 2025^9^ | YES | UNCLEAR | UNCLEAR | YES | UNCLEAR | YES | YES | YES | NO | UNCLEAR | High Risk |

Supplementary table S6: Risk of bias assessment for single-arm studies using JBI Critical Appraisal Checklist for Case Series

Q: Question of Joanna Briggs Institute questionnaire

| Study | Randomization process | Deviations from planned interventions | Missing outcome data | Measurement of the outcome | Selection of reported result | Overall Risk of Bias |
| --- | --- | --- | --- | --- | --- | --- |
| Saito, 2023^3^ | Low | Low | Low | Low | Low | Low Risk |
| Erlinge, 2024^5^ | Low | Low | Low | Low | Low | Low Risk |
| Tsai, 2026^10^ | Low | Low | Low | Low | Low | Low Risk |

**Supplementary table S7:** Risk of bias assessment for randomized-controlled trials using RoB2

# Supplementary appendix

**Landmark data (6-12 and 6-24 months) for Bioadaptor First-in-Human - Verheye 2020/2022**

For the Bioadaptor First-in-Human study, 2 events occurred within 1 month, outcomes of remaining patients at 1-year were reported as zero events (no cardiovascular death, reinfarction, TLR or stent thrombosis). Landmark 6-12-month values were therefore set to zero; similarly, this applied for landmark 6-24 months.

**Landmark data (6-12 months) reconstruction for Bioadaptor RCT - Saito 2023**

In BIOADAPTOR RCT, 1-year ITT data and 2-year landmark results demonstrated that all TLF and TLF component events up to 12 months occurred within the first 6 months (all TV-MI peri-procedural, both 1-year cardiovascular deaths ≤30 days, early CD-TLR linked to these events). The 6-24-month landmark analysis reported only events occurring beyond 12 months (one CD-TLR in the bioadaptor arm, two cardiovascular deaths and four CD-TLR in the DES arm). Because all reported events occurred within the first 6 months and no additional events were reported between 6 and 12 months, landmark incidences were reconstructed as 0/n (n = 219 vs 214 patients at risk at 6 months in the ITT population).

**Landmark data (6-12 months) for New-Zealand registry - Webster 2024**

For the New-Zealand registry, all 1-year outcomes were reported as zero events (no cardiovascular death, reinfarction, TLR or stent thrombosis). Landmark 6-12-month values were therefore set to zero, and no 6-24-month reconstruction was performed because follow-up beyond 12 months was not available.

**Landmark data (6-24 months) reconstruction for INFINITY-SWEDEHEART - Erlinge 2025**

For the 6-24-month landmark interval, the trial group reported cumulative event rates and the corresponding landmark risk-set denominators, but not absolute counts. To allow comparison with other studies, event numbers for TLF and its components were derived by multiplying the reported percentages by the landmark population (1165 vs 1178 patients) and rounding to the nearest whole number, consistent with recommended methods for reconstructing count data from reported percentages. These values are treated as reconstructed estimates from trial-reported percentages rather than primary tabulated outcomes and are interpreted accordingly.

**Landmark data (6-12 months) for Hong-Kong registry - Yan 2023**

The Hong Kong Registry reported no deaths, MI, TLR or stent thrombosis through 12 months. Zero-event rates were extracted as reported. No data beyond 12 months were available; therefore, 6–24-month landmark outcomes were not derived.

**Landmark data (6-12 months and 6-24 months) for DYNAMITE - Leone 2026**

For DYNAMITE, 6-12-month and 6-24-month landmark outcomes were reconstructed from the reported Kaplan-Meier curves and manuscript text, which indicated no additional clinical events beyond 12 months. All primary endpoint events were described by the authors and therefore, event counts at 24 months are identical to those at 12 months.

**Landmark data (6-12 months) for Singapore STEMI - Ho 2025**

For the Singapore STEMI 2 registry, all 1-year outcomes were reported as zero events (no cardiovascular death, reinfarction, TLR or stent thrombosis). Landmark 6-12-month values were therefore set to zero, and no 6-24-month reconstruction was performed because follow-up beyond 12 months was not available.

**Landmark data (6-12 months) for Hong-Kong Single-center registry (United Christian Hospital) - Wong 2025**

The authors reported all adverse events that occurred within one year of follow up. Two major adverse cardiac events occurred within 30 days and no further adverse events were reported through one year follow-up. Therefore, we extracted landmark data with 40 patients at risk at 6 months.

**Landmark data (6-12 months) for Malaysia Single-center registry (Hospital Sultanah Bahiyah, Malaysia) - Kang 2025**

The authors reported all adverse events that occurred within one year of follow up. One target-lesion failure occurred within 30 days and no further adverse events were reported through one year follow-up. Therefore, we extracted landmark data with 24 patients at risk at 6 months.

**Landmark data (6-12 months) for DC3 trial (Dynamic Change of Coronary Artery Curvature) - Tsai 2026**

For the DC 3 single-blind RCT, all 1-year outcomes were reported as zero events (no cardiovascular death, reinfarction, TLR or stent thrombosis). Landmark 6-12-month values were therefore set to zero, and no 6-24-month reconstruction was performed because follow-up beyond 12 months was not available.

**Search strategies**

### PubMed

(DynamX[tiab] OR "DynamX bioadaptor"[tiab] OR bioadaptor[tiab] OR "coronary bioadaptor"[tiab] OR "coronary bioadaptor system"[tiab] OR "sirolimus-eluting bioadaptor"[tiab] OR "DynamX sirolimus"[tiab] OR "Elixir DynamX"[tiab])

### Embase via ovid

(dynamx.ti,ab. OR "dynamx bioadaptor".ti,ab. OR bioadaptor.ti,ab. OR "coronary bioadaptor".ti,ab. OR "coronary bioadaptor system".ti,ab. OR "sirolimus-eluting bioadaptor".ti,ab. OR "elixir dynamx".ti,ab. OR "dynamx sirolimus".ti,ab.) {No Related Terms}

### CENTRAL

("DynamX" OR "DynamX Bioadaptor" OR "Elixir DynamX")

### Google Scholar

("DynamX bioadaptor" OR "coronary bioadaptor" OR "coronary bioadaptor system" OR "sirolimus-eluting bioadaptor" OR "DynamX sirolimus" OR "Elixir DynamX") AND (coronary OR "percutaneous coronary" OR PCI OR "acute coronary syndrome" OR ACS OR "myocardial infarction" OR STEMI OR NSTEMI)
